# β-Amyloid induces microglial expression of GPC4 and APOE leading to increased neuronal tau pathology and toxicity

**DOI:** 10.1186/s13024-025-00883-4

**Published:** 2025-08-29

**Authors:** Brandon B. Holmes, Thaddeus K. Weigel, Jesseca M. Chung, Sarah K. Kaufman, Brandon I. Apresa, James R. Byrnes, Kaan S. Kumru, Jaime Vaquer-Alicea, Ankit Gupta, Indigo V. L. Rose, Yun Zhang, Alissa L. Nana, Dina Alter, Lea T. Grinberg, Salvatore Spina, Kevin K. Leung, Bruce L. Miller, Carlo Condello, Martin Kampmann, William W. Seeley, Jaeda C. Coutinho-Budd, James A. Wells

**Affiliations:** 1 Department of Neurology, University of California, San Francisco, San Francisco, CA 94158 USA; 2 Weill Institute for Neurosciences, University of California, San Francisco, San Francisco, CA 94158 USA; 3 Department of Neuroscience, University of Virginia School of Medicine, Charlottesville, VA 22903 USA; 4 Department of Pharmaceutical Chemistry, University of California, San Francisco, San Francisco, CA 94158 USA; 5 Center for Alzheimer’s and Neurodegenerative Diseases, Peter O’Donnell Jr. Brain Institute, University of Texas Southwestern Medical Center, Dallas, TX 75235 USA; 6 Institute for Neurodegenerative Diseases, University of California, San Francisco, San Francisco, CA 94158 USA; 7 Neuroscience Graduate Program, University of California, San Francisco, San Francisco, CA 94158 USA; 8 Department of Pathology, University of California, San Francisco, San Francisco, CA 94158 USA; 9 Department of Biochemistry and Biophysics, University of California, San Francisco, San Francisco, CA 94158 USA; 10 Department of Cellular and Molecular Pharmacology, University of California, San Francisco, San Francisco, CA 94158 USA

**Keywords:** Alzheimer’s disease, Microglia, GPC4, Heparan sulfate proteoglycans, Amyloid, Tau seeding, APOE, *Drosophila* model, Neuroinflammation, Astrocytes

## Abstract

To define how Aβ pathology alters microglia function in Alzheimer’s disease, we profiled the microglia surfaceome following treatment with Aβ fibrils. Our findings reveal that Aβ-associated human microglia upregulate Glypican 4 (GPC4), a GPI-anchored heparan sulfate proteoglycan (HSPG). Glial GPC4 expression exacerbates motor deficits and reduces lifespan in a *Drosophila* amyloidosis model, implicating GPC4 in a toxic neurodegenerative program. In cell culture, GPC4 enhances microglia phagocytosis of tau aggregates, and shed GPC4 can act *in trans* to facilitate tau aggregate uptake and seeding in neurons. Additionally, our data demonstrate that GPC4-mediated effects are amplified in the presence of APOE. In human Alzheimer’s disease brain, microglial GPC4 expression surrounding Aβ plaques correlates with neuritic tau pathology, supporting a pathological link between amyloid, GPC4, and tau. These studies define a mechanistic pathway by which Aβ primes microglia to promote tau pathology via HSPGs and APOE.

**Supplementary Information:**

The online version contains supplementary material available at 10.1186/s13024-025-00883-4.

## Background

The defining pathological features of Alzheimer’s disease are the accumulation of β-amyloid (Aβ) plaques and tau neurofibrillary tangles [1, 2]. Rodent and human studies suggest that Aβ accelerates the propagation of tau pathology within brain networks, likely through both local and remote amyloid-tau interactions [3–8]. Indeed, the anti-Aβ monoclonal antibodies, lecanemab and donanemab, reduce the deposition of tau pathology in patients with Alzheimer’s disease (AD), possibly via the removal of upstream amyloid plaques [9–11]. However, the cellular and molecular mechanisms by which Aβ incites the spread of tau pathology remain unknown.

As the resident immune cells of the brain, microglia are the frontline reporters and executioners of inflammatory activity and injury response in neurodegeneration. Emerging evidence now links microglia biology to various neurodegenerative disorders, including AD. Multi-omics analyses – including GWAS, gene expression data, epigenetic annotations, cellular pathway analyses, and cell-type specific promoter mapping – show that many common AD risk variants are either expressed uniquely in microglia or enriched in microglia-specific enhancer elements [12–15]. Further, integrative network analyses demonstrate that AD genetic variants converge on microglia biological processes such as phagocytosis, endolysosomal processing, and lipid metabolism [16, 17]. Thus, a substantial body of genetic data implicate a central role for microglia in the pathogenesis of AD.

Emerging experimental data suggest that reactive microglia contribute to the deposition of pathological tau as well as tau-mediated neurodegeneration in AD [18–21], possibly via APOE [22, 23]. Human PET imaging studies demonstrate that tau propagation pathways depend on microglia networks, and the co-occurrence of Aβ, tau, and microglia reactivity is a stronger predictor of cognitive impairment than the combination of Aβ and tau alone [24]. Given that microglia reactivity may precede tau pathology [25, 26], we hypothesize that AD reactive microglia are *primed* to accelerate pathological tau spread and that this priming may result from Aβ pathology.

To interrogate how microglia respond to Aβ, we profiled the iPSC-derived microglia surfaceome with surface biotinylation using WGA-HRP glycoproteomics [27] after treatment with recombinant Aβ fibrils. We found that Aβ stimulates microglia to remodel their cell surface and specifically express Glypican 4 (GPC4), a GPI-anchored heparan sulfate proteoglycan not previously implicated in microglia biology. In AD patients, microglia associated with Aβ plaques upregulate GPC4, and in vivo, glial GPC4 expression accelerates amyloid-related toxicity. The expression of GPC4 enhances tau aggregate uptake in microglia and neurons, and, in concert with APOE, potentiates intraneuronal tau seeding and pathology. Consistent with this model, we show that in human AD brain microglial GPC4 expression surrounding Aβ plaques strongly correlates with neuritic tau pathology. This observation supports the idea that Aβ-associated microglial states influence the local development of aggregated tau in AD. These findings motivate the development of novel microglia-targeted therapeutic interventions to disrupt Aβ-induced toxicity and spread of tau pathology.

## Results

### iTF Microglia surfaceomics reveals Aβ fibril-induced upregulation of heparan sulfate proteoglycans and glypicans

The pathology of AD elicits a profound microglial reaction, generating unique transcriptional and functional microglial features [28, 29]. Since microglia are highly dynamic cells capable of altering their receptor profiles in response to tissue injury [30, 31], we aimed to investigate Aβ-associated surfaceome remodeling. We performed cell surface proteomics on human iPSC-derived induced-transcription factor microglia (iTF Microglia) [32]. The iTF Microglia recapitulate core microglia biology, as assessed by the expression of microglia markers and functional responses to lipopolysaccharide (LPS) and phagocytic substrates (Fig. S1A – E). Given that microglia rapidly react to their microenvironment with altered protein expression, we employed an efficient surfaceome labeling strategy previously developed by our lab [27]. This technology uses horseradish-peroxidase tethered to wheat-germ agglutinin (WGA-HRP) to bind the cell surface and biotinylate resident membrane proteins.

**Fig. 1:**
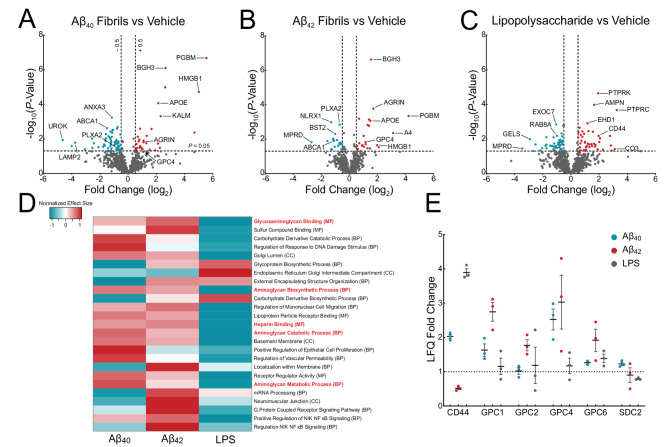
Surfaceomics of iTF Microglia reveal upregulation of glypicans. Volcano plots showing changes in surface proteins from iTF Microglia treated with (**A**) 1 µM Aβ_40_ fibrils, (**B**) 1 µM Aβ_42_ fibrils, or (**C**) 100 ng/mL LPS. The -log_10_ transformed p-value is plotted against the log_2_ transformed label-free quantitation ratios (log_2_ fold changes). *N* = 3–6 biological replicates. (**D**) Gene Set Enrichment Analysis of iTF Microglia treated with Aβ_40_ fibrils, Aβ_42_ fibrils, or LPS. The heatmap shows the normalized effect size of the top 25 most statistically altered pathways. Positive normalized effect size is shown in red (upregulation), and negative normalized effect size is shown in blue (downregulation). Gene Ontology terms highlighted in red font relate to glycosaminoglycan and proteoglycan biology. (**E**) Mass spectrometry label-free quantitation of cell surface heparan sulfate proteoglycans found in the surfaceomics experiments (**A**, **B**, **C**).

To elucidate AD-associated surfaceome changes, we treated iTF Microglia with recombinant Aβ_40_ fibrils, Aβ_42_ fibrils (Fig. S1F) or lipopolysaccharide (LPS) for 72 h to allow surfaceome remodeling. LPS was used to elicit a generic innate immune response, providing a comparison to amyloid-specific proteomic changes. Compared to vehicle-treated iTF Microglia, Aβ_40_ fibrils resulted in the significant upregulation (> 1.5-fold) of 30 proteins and downregulation of 62 proteins (Fig. 1A); Aβ_42_ fibrils led to the significant upregulation of 28 proteins and downregulation of 27 proteins (Fig. 1B). Of the differentially expressed proteins, 13 (9.7%) were shared between Aβ_40_ and Aβ_42_ fibril-treated iTF Microglia (Fig. S2A). LPS led to the upregulation of 46 proteins and downregulation of 47 proteins (Fig. 1C). Only 8 (5.42%) and 11 (8.03%) of these differentially expressed proteins were shared between Aβ_40_ and Aβ_42_ fibril-treated iTF Microglia, respectively; and only 3 (2.8%) proteins were shared between all three treatments (Fig. S2A). Principal component analysis demonstrated distinct clustering of Aβ_40_-, Aβ_42_-, and LPS-treated microglia compared to vehicle controls, indicating specific remodeling of the surfaceome (Fig. S2B).

To evaluate for thematic surfaceome changes, we employed Gene Set Enrichment Analysis (Fig. 1D and S2C). Among the top 25 most statistically significant Gene Ontology terms, five implicated a role in proteoglycan biology, including glycosaminoglycan binding (GO:0005539), aminoglycan biosynthetic process (GO:0006023), aminoglycan catabolic process (GO:0006027), aminoglycan metabolic process (GO:0006022), and heparin binding (GO:0008201). Additionally, STRING network and UniProt keyword enrichment analyses highlighted the involvement of pathways related to proteoglycans and extracellular matrix remodeling, heparan sulfate, lipid metabolism, endocytosis, and Alzheimer’s disease (Fig. S2D and S2E). Within the proteins comprising these groups of Gene Ontology terms (Fig. 1D and S2C), our mass spectrometry identified 8 proteoglycans on iTF Microglia, six of which were cell surface bound (Fig. 1E), and four of which were in the glypican family of heparan sulfate proteoglycans (HSPGs). These glypican HSPGs, particularly GPC4, were enriched in the Aβ_40_ and Aβ_42_ fibril-treated iTF Microglia but not in the LPS-treated cells suggesting that their expression may be specifically induced by amyloid pathology. A role for glypicans in microglia has not been previously defined in health or disease. The full list of differentially expressed surface proteins for all treatment comparisons, along with their log_2_-transformed LFQ intensities, Z-scores, log_2_ fold changes and FDR-adjusted p-values, is provided in Supplementary Material S13.

### iTF Microglia uniquely and specifically upregulate heparan sulfate and GPC4 in response to Aβ fibrils

**Fig. 2:**
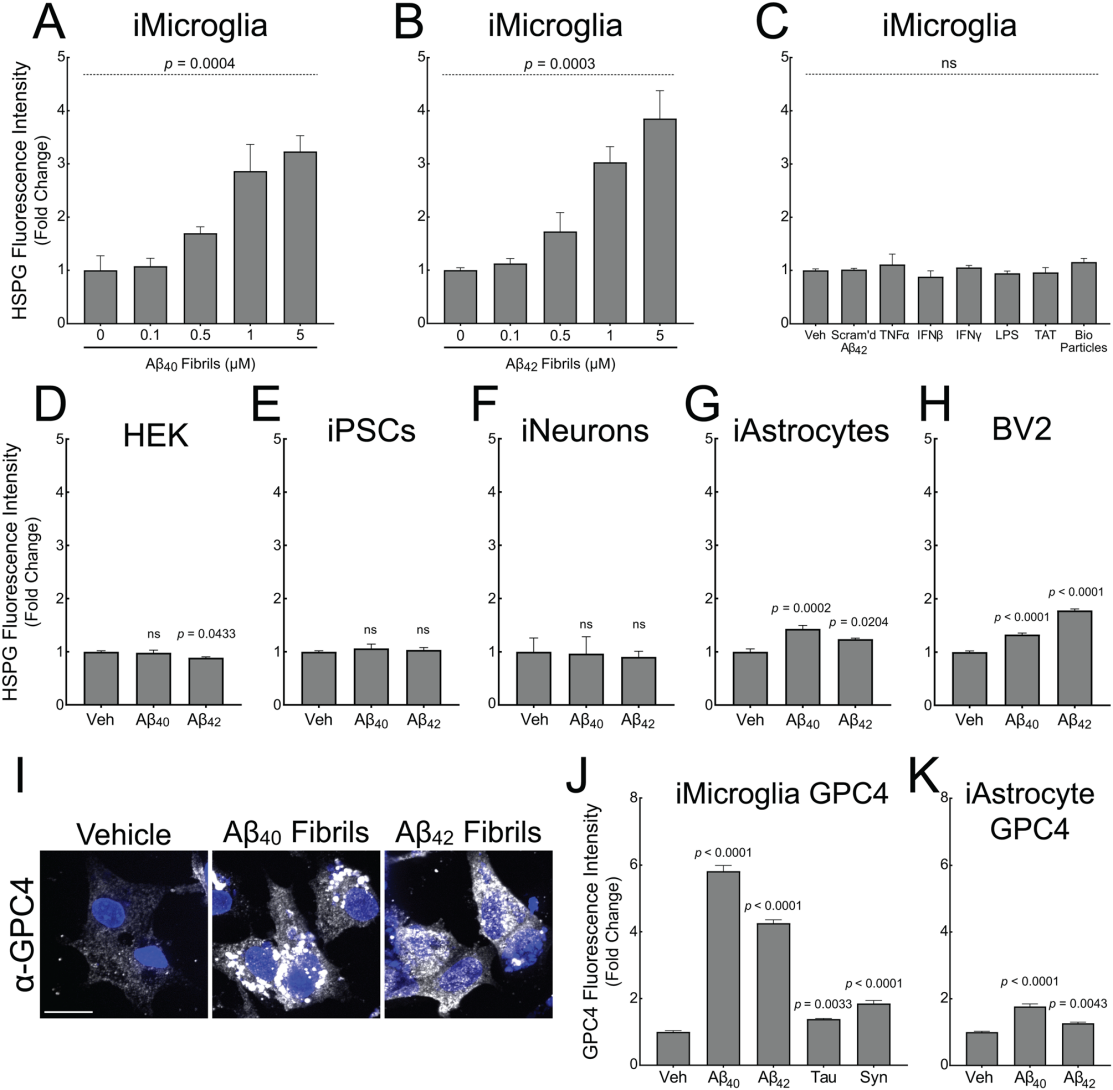
Amyloids induce iTF Microglial cell surface heparan sulfate and GPC4 expression. iTF Microglia treated with (**A**) Aβ_40_ and (**B**) Aβ_42_ fibrils have dose-dependent increases in cell surface heparan sulfate as measured by flow cytometry with 10E4 antibody. (**C**) Other inflammatory substrates and controls do not alter cell surface heparan sulfate levels. The statistical analyses were performed with a one-way ANOVA. *N* = 3 biological replicates. Heparan sulfate cell surface staining with 10E4 on (**D**) HEK293T cells, (**E**) iPSCs, (**F**) iNeurons, (**G**) iAstrocytes, and (**H**) BV2 cells treated with 1 µM Aβ_40_ and Aβ_42_ fibrils. The statistical analyses were performed with a one-way ANOVA and Holm-Sidak multiple comparisons tests for the adjusted *p*-values. *N* = 3 biological replicates. (I) GPC4 immunocytochemistry of iTF Microglia treated with 1 µM Aβ_40_ or Aβ_42_ fibrils. Scale bar = 10 μm. GPC4 flow cytometry quantification of (**J**) iTF Microglia or (**K**) iAstrocytes treated with 1 µM proteopathic amyloid fibrils. The statistical analyses were performed with a one-way ANOVA and Holm-Sidak multiple comparisons tests for the adjusted *p*-values. *N* = 3 biological replicates. In all graphs, the data represent the means ± SEM.

HSPGs have been previously recognized to co-deposit within extracellular amyloid plaques [33, 34], as well as play a role in tau protein aggregate uptake and intracellular seeding in neurons [35, 36, 37]. To further explore the involvement of HSPGs and glypicans in AD-related microglia, we directly tested whether Aβ fibrils can increase cell surface heparan sulfate in microglia. iTF microglia treated with Aβ_40_ or Aβ_42_ fibrils for 24 h showed a dose-dependent increase, up to four-fold, in cell surface heparan sulfate as measured by flow cytometry using an anti-HSPG antibody (clone 10E4) (Fig. 2A and B). Importantly, treatment with Aβ_42_ monomer did not result in increased cell surface heparan sulfate (Fig. S3A) suggesting that amyloids, but not cognate monomer, have the capacity to induce cell surface HSPGs. Furthermore, iTF Microglia treated with innate immune activators TNFα (1 nM), IFNβ (100 pM), IFNγ (100 pM), LPS (100 ng/mL), BioParticles (10 µg/mL), the heparan sulfate-binding TAT peptide (5 µM), or scrambled Aβ_42_ peptide for 24 h also did not increase cell surface heparan sulfate (Fig. 2C) suggesting that generic inflammation is insufficient to increase microglial HSPGs. To determine if microglia increase their heparan sulfate in response to other amyloids, we treated iTF Microglia with tau monomer, synuclein monomer, tau fibrils, or synuclein fibrils (Fig. S1F) and again quantified heparan sulfate. Both tau fibrils and synuclein fibrils, but not cognate monomers, induced the expression of cell surface heparan sulfate further confirming that amyloids, but not their monomeric equivalent, are competent to elicit this HSPG response (Fig. S3A).

We next investigated whether Aβ fibrils can prime other cell types to upregulate cell surface heparan sulfate. We treated HEK293T cells, undifferentiated iPSCs (precursors to iTF Microglia), iNeurons, iAstrocytes, and mouse BV2 cells with 1 µM Aβ_40_ fibrils or Aβ_42_ fibrils for 24 h. HEK293T cells, iPSCs, and iNeurons did not increase cell surface heparan sulfate by 10E4 staining (Fig. 2D and F). iAstrocytes and BV2 cells responded to Aβ fibrils with a modest (< 2-fold) increase in cell surface heparan sulfate (Fig. 2G and H). To assess whether this altered heparan sulfate response relates to the capacity of these cells to internalize Aβ fibrils, we next treated each cell line with 1 µM pHrodo red-labeled Aβ_42_ fibrils for 24 h and measured uptake by microscopy. iTF Microglia exhibited the highest uptake of Aβ fibrils followed by BV2 cells (93.8% of iTF Microglia). HEK293T cells showed moderate uptake (40.7% of iTF Microglia), while iAstrocytes (10.6%), iNeurons (0.6%), and iPSCs (1.2%) showed the lowest levels (Fig. S3B). Across cell types, there was a statistically significant correlation between Aβ_42_ fibril uptake and the degree of HSPG upregulation (Fig. S3C), suggesting that amyloid internalization may be a contributing factor in cell surface heparan sulfate remodeling. However, the relationship was not strictly monotonic, indicating that additional cell-intrinsic factors may modulate the HSPG response to amyloid exposure. These findings suggest that human microglia may be particularly responsive to Aβ-induced heparan sulfate expression.

Cell surface heparan sulfates are known to be attached to glypican (GPC1–6) and syndecan (SDC1–4) core protein family members. From our iTF Microglia surfaceomics, we identified four out of six glypican family members. GPC4, in particular, was significantly upregulated upon Aβ_42_ fibril treatment. To test if Aβ fibrils directly upregulate cell surface glypicans, we treated iTF microglia with 1 µM Aβ_40_ or Aβ_42_ fibrils for 24 h. Aβ_40_ and Aβ_42_ fibrils robustly elevated GPC4 expression as measured by microscopy, flow cytometry, and qPCR whereas tau fibrils and synuclein fibrils only modestly elevated GPC4 (Fig. 2I and J and S3D). Moreover, the amount of Aβ fibril phagocytosis correlated with the degree of GPC4 upregulation on a per-cell basis in iTF Microglia (Fig. S4A and B). Further, Aβ fibril treatment led to minimal increases in iAstrocyte GPC4 (Fig. 2K). These data support the concept that microglia, more so than astrocytes, undergo Aβ-induced GPC4 expression, which may be partially explained by the degree of Aβ engulfment. Finally, we evaluated the effect of Aβ fibril treatment on iTF Microglia cell surface expression for the remainder of the glypican family. GPC2, GPC3, and GPC5 were weakly expressed in iTF Microglia and unchanged after treatment with Aβ fibrils (Fig. S3E). GPC6 expression was elevated after treatment with Aβ fibrils, albeit to a much smaller degree than GPC4 (Fig. S3E and F). We could not detect the presence of GPC1 (data not shown). Taken together, this data demonstrate that recombinant Aβ fibrils robustly and specifically upregulate microglial GPC4 expression in the iPSC culture system.

### Human microglial GPC4 expression correlates with Aβ plaque pathology in Alzheimer’s disease

**Fig. 3:**
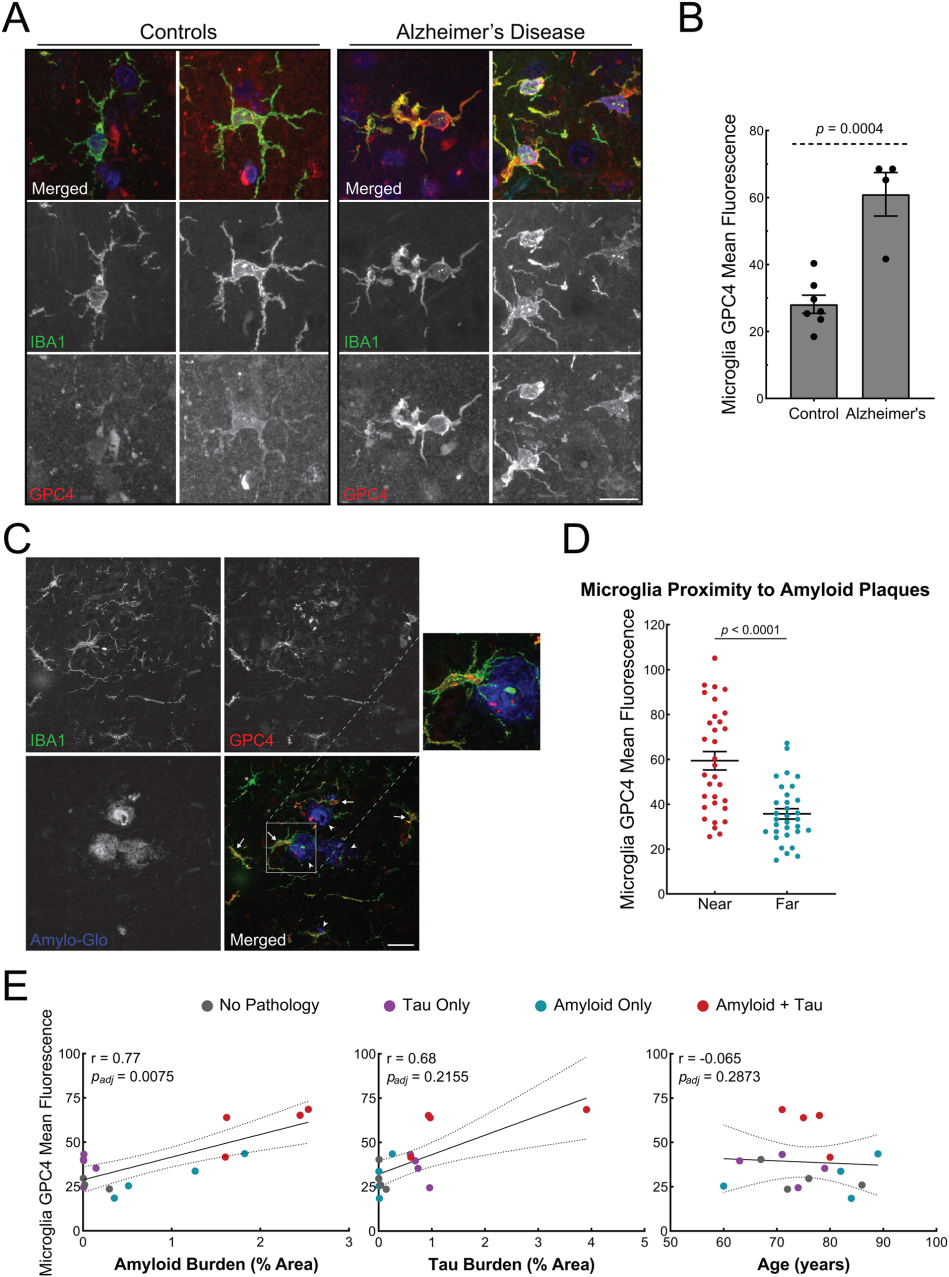
Microglial GPC4 expression is upregulated in human AD brain and correlates with amyloid pathology. (**A**) Representative confocal images of IBA1 (green), GPC4 (red), and DAPI (blue) in two age-matched controls and two AD cases. Scale bar = 20 μm. (**B**) GPC4 mean fluorescence intensity measurements in IBA1^+^ microglia from age-matched controls (*N* = 7) and AD (*N* = 4) cases. The statistical analysis was performed with a Student t-test for averaged values from individual subjects. The data represent the means ± SEM. (**C**) Representative confocal images of IBA1 (green), GPC4 (red), and Amylo-Glo (blue) in an AD case. Arrowhead = amyloid plaque; Arrow = GPC4^+^ microglia; Asterisk = GPC4^−^ microglia. Scale bar = 40 μm. (**D**) Quantification of GPC4 mean intensity values in IBA^+^ microglia located at two distances from Amylo-Glo^+^ Aβ plaques. A total of eight plaques were measured from each brain, and for each plaque, microglia were binned into two separate categories, “near” or “far”, based on proximity to the plaque. Near: < 125 μm from center of plaque; Far: >150 μm and < 350 μm from edge of plaque. The *p*-values were determined by a paired t-test. The data represent the means ± SEM. (**E**) Scatter plots of microglial GPC4 mean fluorescence intensity per each patient versus their amyloid plaque burden, neurofibrillary tangle burden, or age. Pathological categories colored grey for control, purple for individuals for tau pathology only, blue for individuals with Aβ pathology only, or red for individuals with both tau and Aβ pathology. Normal distribution was assessed using the D’Agostino & Pearson test which indicated that GPC4 values, pathological burden values, and age were normally distributed. The *p*-values were determined by Pearson’s correlation with 95% confidence bands and adjusted for confounding by applying multiple linear regression.

To investigate whether microglia express GPC4 in neurodegeneration, we performed immunohistochemistry on human brain sections containing Aβ plaques and neurofibrillary tangles (Alzheimer’s disease), neurofibrillary tangles only (corticobasal degeneration), Aβ plaques only, or neither (healthy age-matched control tissue) (Supplementary Material 14). We stained human tissue with IBA1 and GPC4 antibodies and measured microglial GPC4 expression. Aged-matched control microglia expressed minimal GPC4 whereas AD microglia robustly expressed GPC4 (Fig. 3A). Quantification of microglia-specific expression of GPC4 revealed a 2.2-fold increase in AD samples (Fig. 3B). Additionally, we observed some punctate and low-intensity GPC4 expression in non-microglia cells.

We next investigated whether microglia that are near amyloid plaques express greater levels of GPC4 relative to microglia that are far from plaques. We stained brain sections with IBA1 and GPC4 antibodies as well as Amylo-Glo, a fluorescent dye that labels amyloid plaques (Fig. 3C) [38]. For each AD brain, we selected 8 Amylo-Glo-positive plaques and binned microglia into two groups: near microglia (< 125 µm from the center of the index plaque) or far microglia (> 150 μm from the edge of all plaques and < 350 μm from edge of the index plaque). Microglia near Aβ plaques had significantly higher GPC4 expression compared to microglia far from plaques, suggesting that close proximity to amyloid plaques correlates with higher microglial GPC4 levels (Fig. 3D). Notably, this trend persisted in human brains containing Aβ plaque pathology without neurofibrillary tangles suggesting that microglial GPC4 expression is independent of tau pathology (Fig. S5A).

We next assessed whether microglial GPC4 expression correlated with amyloid plaque burden or neurofibrillary tangle burden after staining the tissue with an anti-Aβ antibody (DE2) or anti-phosphorylated tau antibody (CP13). After adjusting the GPC4-amyloid pathology correlation for tau pathology as a confounder by applying multiple linear regression, there was a significant relationship between GPC4 expression and amyloid pathology (*r* = 0.77; *p* = 0.0075), but not for tau pathology (*r* = 0.68; *p* = 0.2155). There was no correlation between microglial GPC4 expression and patient age (Fig. 3E).

Finally, we investigated whether other members of the glypican family were upregulated in AD microglia. We found that GPC1, GPC2, and GPC6 were globally upregulated in human AD tissue compared to healthy aged-matched control. However, GPC1, GPC2, and GPC6, were not expressed by microglia (Fig. S5B), but rather expressed in cells with a neuronal morphology. Taken together, our data indicate that human AD microglia express GPC4 which is correlated with and proximal to Aβ pathology.

### Mouse microglia do not upregulate GPC4 in models of amyloidosis

We next investigated whether mouse models of amyloidosis recapitulate this phenotype. We performed immunohistochemistry to examine microglial GPC4 expression within the frontal lobe cortex and dentate gyrus of aged non-transgenic C57BL/6J, 5xFAD, and App-SAA knock-in mice. Surprisingly, neither of the amyloid models differed from non-transgenic controls in terms of GPC4 expression in IBA1^+^ microglia (Fig. S6A–E). However, punctate and intense GPC4 expression was detected in GFAP^+^ astrocytes, but this expression was not significantly different between non-transgenic and transgenic mice. These data demonstrate that mouse models of amyloidosis do not recapitulate human microglial GPC4 expression, but rather display persistent astrocyte expression in adulthood. Recent research reveals significant evolutionary divergence between rodent and primate microglia with human microglia exhibiting the greatest transcriptional heterogeneity and number of cellular states [39, 40]. This implies that rodent microglia may have a distinct reactivity profile in comparison to humans, although current rodent studies have not directly compared disease-relevant conditions. Therefore, it is possible that the human upregulation of microglial GPC4 in AD is yet another example of evolutionary divergence between rodents and primates.

### Glial GPC4 enhances toxicity in a *Drosophila* model of amyloidosis

**Fig. 4:**
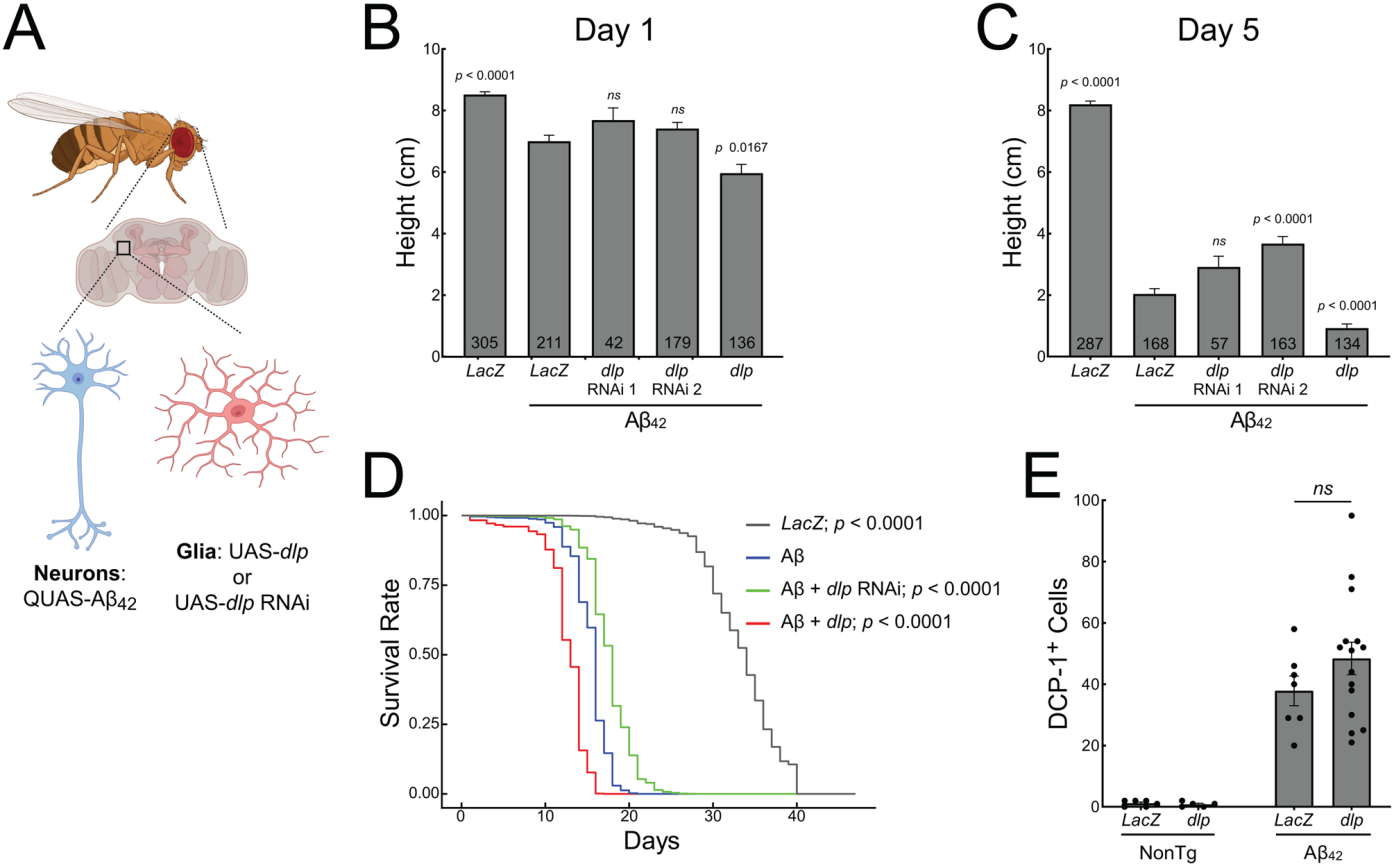
Glial GPC4 worsens climbing and early lethality in an amyloid model of *Drosophila*. (**A**) Schematic model depicting *Drosophila* transgenic lines in which neurons express Aβ_42_ via the QUAS promoter and glia express *dlp* or *dlp* RNAi via the UAS promoter. Climbing heights were measured at day 1 post-eclosion (**B**) or day 5 post-eclosion (**C**) in Aβ_42_ flies expressing LacZ, dlp RNAi, or dlp cDNA. The numbers within the bars represent the number of flies measured per condition. The statistical analyses were performed with a Tobit regression with Bonferroni correction. (**D**) Median fly lifespans measured in days after eclosion (LacZ = 34 d; Aβ_42_ = 16 d; Aβ_42_ + *dlp* RNAi = 18 d; Aβ_42_ + *dlp* = 12 d). Lifespan data was analyzed using a Cox proportional hazard model with Bonferroni corrections. (LacZ *n* = 174; Aβ_42_*n* = 134; Aβ_42_ + *dlp* RNAi *n* = 56; Aβ_42_ + *dlpn* = 102). (**E**) *Drosophila* brains were immunostained for cleaved Dcp-1 and positive cells were counted across the entire brain. The statistical analysis was performed with a Student t-test. The error bars represent the SEM values.

Since microglial GPC4 expression is not observed in rodent models of amyloidosis, we employed *Drosophila melanogaster* to directly manipulate the glial expression of *Dlp*, the fly ortholog of human GPC4. We first investigated whether glial expression of *Dlp* modifies amyloid toxicity in vivo by using a previously established transgenic *Drosophila* model of amyloidosis [41] (Fig. 4A and S7A). Neuronal expression of human Aβ_42_ led to profound and progressive climbing deficits, a measure of motor dysfunction (Fig. 4B and C), as well as premature lethality (Fig. 4D). Glial over-expression of *Dlp* progressively worsened climbing at day-one and day-five post-eclosion (Fig. 4B and C) as well as reduced lifespan (Fig. 4D) beyond what was observed for Aβ_42_ alone. Conversely, knockdown of glial *Dlp* with RNAi significantly improved climbing ability (Fig. 4B and C) and extended survival in Aβ_42_-expressing flies (Fig. 4D). The expression of human Aβ_42_ did not alter the expression of endogenous fly *Dlp* as measured by qPCR (Fig. S7B). Although glial *Dlp* knockdown improved Aβ_42_-induced phenotypes, qPCR showed only a trending, non-significant decrease in *Dlp* mRNA, likely because the glial driver targets only a subset of cells while qPCR was performed on bulk brain RNA (Fig. S7B). Further, the effect of *Dlp* was amyloid-specific, as these phenotypes were not observed to the same degree in flies lacking human Aβ_42_ expression (Fig. S7C–E).

We next asked whether the toxicity conferred by *Dlp* over-expression is associated with apoptotic cell death. We stained transgenic *Drosophila* brains at 10 days post eclosion with an anti-DCP-1 antibody to detect activated caspase. No significant increase in apoptosis was observed in flies over-expressing glial *Dlp* (Fig. 4E) suggesting that *Dlp* potentiates Aβ toxicity via mechanisms independent of caspase-mediated cell death. Taken together, these data demonstrate that glial-derived *Dlp*, the ortholog of human GPC4, enhances Aβ-induced neurotoxicity in vivo.

### GPC4 mediates tau phagocytosis in iTF Microglia

**Fig. 5:**
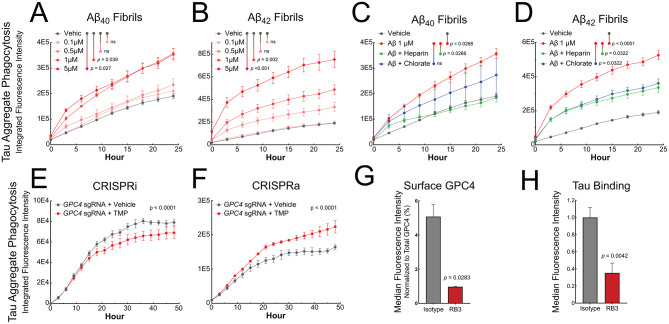
Heparan sulfate and GPC4 mediate tau phagocytosis in iTF Microglia. Phagocytosis of pHrodo red-labeled tau fibrils after pretreatment with Aβ_40_ (**A**) or Aβ_42_ (**B**) fibrils by iTF Microglia. Phagocytosis of pHrodo red-labeled tau fibrils in the presence of heparan sulfate proteoglycan inhibitors heparin (100 µg/mL) or chlorate (25 mM) after pretreatment with Aβ_40_ (**C**) or Aβ_42_ (**D**) fibrils. The statistical analysis for experiments A–D were performed with a one-way ANOVA and Holm-Sidak multiple comparisons test. *N* = 4. Phagocytosis of pHrodo red-labeled tau fibrils using inducible CRISPRi (**E**) or inducible CRISPRa (**F**) iTF Microglia transduced with GPC4 sgRNAs. The CRISPRa and CRISPRi elements are activated by trimethoprim (50 nM). The statistical analyses for experiments E, F were performed with a paired t-test. *N* = 4. (**G**) Flow cytometry analysis of iTF Microglia measures the abundance of cell surface GPC4, normalized to total GPC4 levels, after treatment with α-GPC4 sdAb-Fc or isotype control antibodies for 24 h. (**H**) Flow cytometry analysis of AF647-labeled tau fibrils binding to the iTF Microglia cell surface after pre-treatment with an α-GPC4 antibody. The cell surface binding experiment was performed at 4 °C. The statistical analyses were performed with a Student t-test. The data represent the means ± SEM.

Having confirmed that GPC4 is upregulated in human AD-associated microglia and potentiates toxicity in vivo, we next investigated the impact of GPC4 on microglial function. Our prior work in neurons demonstrates that HSPGs govern the internalization and propagation of tau pathology [35, 36, 42]. However, the specific core proteins mediating this response remain unknown. Here, we hypothesized that Aβ fibril-induced GPC4 expression may facilitate tau aggregate phagocytosis in microglia. To investigate this, we primed iTF microglia with varying concentrations of Aβ_40_ or Aβ_42_ fibrils for 24 h, washed the cells, applied pHrodo-labeled tau fibrils, and monitored phagocytosis over time. We observed a significant increase in tau phagocytosis when iTF Microglia were primed with increasing doses of Aβ fibrils (Fig. 5A and B). This Aβ-induced tau phagocytosis was inhibited by ~ 50% by the co-application of heparin or chlorate, inhibitors of heparan sulfate proteoglycans (Fig. 5C and D) demonstrating that heparan sulfate contributes to Aβ priming of iTF microglia. Aβ priming did not alter the phagocytosis of magnetic beads (Fig. S8A) which are not known to enter microglia in an HSPG-dependent manner. To specifically assess if GPC4 mediates tau aggregate phagocytosis, we employed iTF Microglia cells expressing trimethoprim-inducible CRISPRi and CRISPRa machinery to downregulate and upregulate GPC4, respectively [32]. Downregulation of GPC4 via CRISPRi reduced tau fibril phagocytosis, whereas upregulation of GPC4 via CRISPRa increased phagocytosis (Fig. 5E and F). The extent of tau fibril phagocytosis was proportional to the degree of GPC4 expression change as measured by flow cytometry (Fig. S8C and D).

We next tested whether a GPC4 function-blocking antibody can inhibit tau fibril phagocytosis. RB3 is an anti-GPC4 single domain antibody (sdAb) previously shown to inhibit GPC4 activity and promote differentiation of pluripotent stem cells into midbrain dopaminergic neurons [43]. We grafted RB3 onto a human IgG1 Fc scaffold to create a bivalent anti-GPC4 sdAb-Fc (Fig. S8B). Treatment of iTF Microglia with 100 nM RB3 for 24 h led to ~ 80% depletion of cell surface GPC4 as measured by flow cytometry with a commercial polyclonal antibody (mean ± SD: isotype = 2,844 ± 559 AU; RB3 = 544.5 ± 9.2 AU), presumably due to increased intracellular trafficking and turnover of RB3-bound GPC4 (Fig. 5G). Importantly, total GPC4 levels measured in whole-cell lysates remained unchanged following RB3 treatment (isotype = 56,065 ± 8,948 AU; RB3 = 58,591 ± 9,249 AU), supporting the interpretation that RB3 primarily alters GPC4 surface localization. This antibody treatment also led to a 65% reduction in tau-647 fibril binding to the iTF Microglia surface in a cell-based binding assay at 4 °C (Fig. 5H). Taken together, these data demonstrate that GPC4 mediates tau fibril binding and phagocytosis in iTF Microglia.

### Aβ fibrils lead to GPC4 shedding which promotes tau phagocytosis

**Fig. 6:**
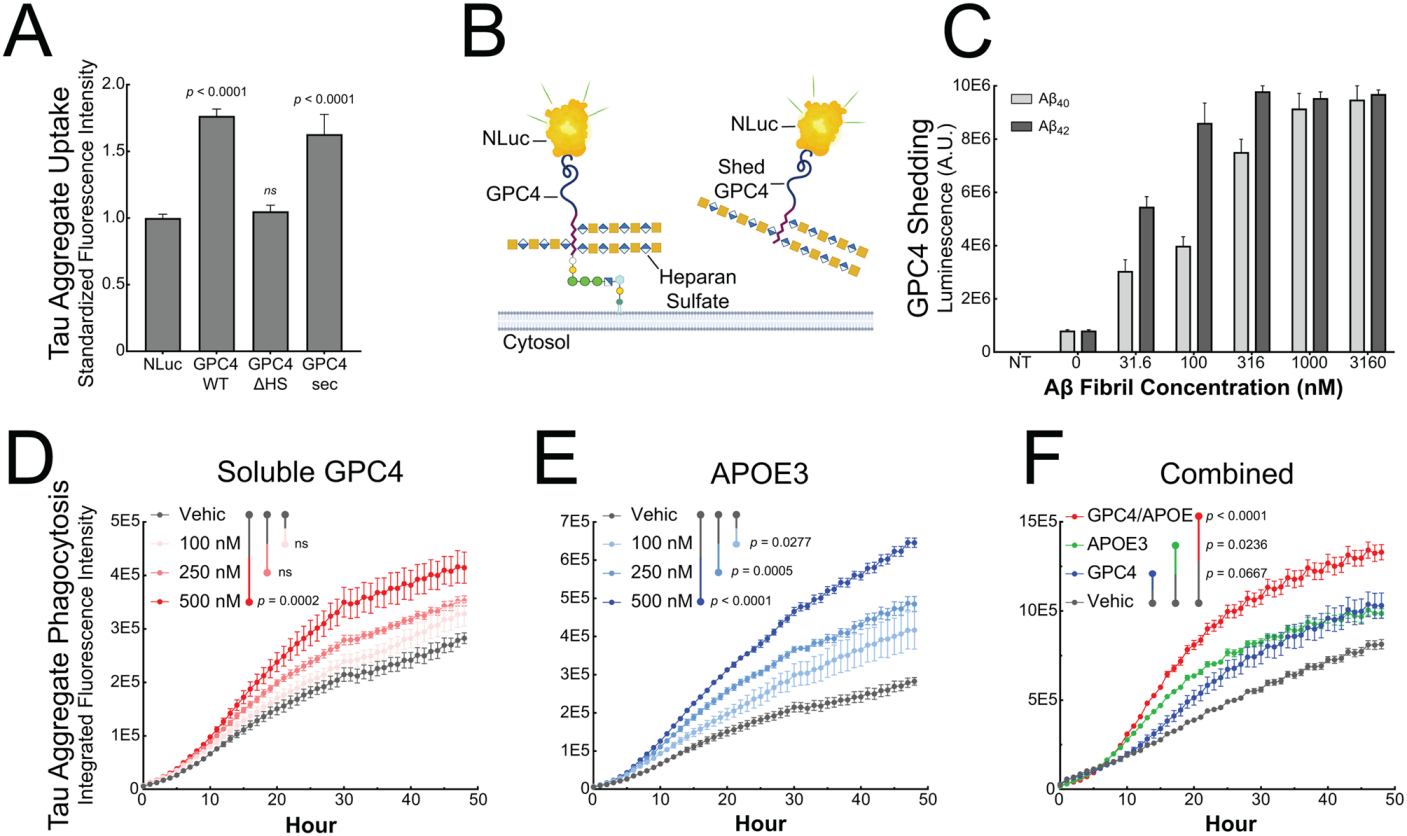
β-Amyloid fibrils lead to GPC4 shedding and APOE secretion which promote tau phagocytosis. (**A**) GPC4 WT, GPC4-ΔHS, GPC4-sec, or NLuc control plasmids were transiently transfected into HEK293T cells and tau aggregate-AF647 uptake was measured via flow cytometry. (**B**) Schematic model depicting NLuc-GPC4 fusion protein for tracking GPC4 shedding into the conditioned media via luminescence. (**C**) NLuc-GPC4 luminescence was measured in iTF Microglia conditioned media after a 24 h treatment with Aβ_40_ and Aβ_42_ fibrils. pHrodo-red labeled tau aggregates (50 nM) were preincubated with (**D**) soluble recombinant GPC4 or (**E**) soluble recombinant APOE3 and uptake was measured every hour for 48 h with an Incucyte SX5 in iTF Microglia. (**F**) pHrodo-red labeled tau aggregates (50 nM) were preincubated with soluble recombinant GPC4 alone, APOE3 alone, or GPC4 + APOE3 (250 nM) and uptake was measured every hour for 48 h in iTF Microglia. The statistical analyses were performed with a one-way ANOVA and Holm-Sidak multiple comparisons test. *N* = 4. The data represent the means ± SEM.

GPC4 is a GPI-anchored HSPG that can exist in multiple proteoforms. To determine which GPC4 proteoform is most bioactive in mediating tau phagocytosis, we created three GPC4 genetic variants that encode (1) the natural cell surface bound GPC4 (GPC4 WT), (2) a GPC4 variant lacking heparan sulfate (HS) chains due to ablated HS attachment sites (GPC4-ΔHS), and (3) a constitutively secreted GPC4 variant that lacks the GPI-anchor (GPC4-sec). We expressed these three variants in HEK cells and mouse microglia BV2 cells and measured their ability to internalize pHrodo-labeled tau fibrils. Over-expression of GPC4 WT enhanced tau fibril internalization by 76% in HEK293T cells and 25% in BV2 cells compared to NanoLuc (NLuc) expression control (Fig. 6A and S9A). These data are consistent with the GPC4 CRISPRa experiment in iTF Microglia (Fig. 5F). In contrast, GPC4-ΔHS failed to augment tau fibril phagocytosis suggesting a critical role for heparan sulfate chains in tau uptake as we previously reported [35]. Surprisingly, we found that the constitutively secreted variant of GPC4, GPC4-sec, was an equally bioactive proteoform, increasing tau fibril internalization by 63% in HEK293T cells and 36% in BV2 cells. (Fig. 6A and S9A). This suggests that both the membrane bound and soluble forms of GPC4 are bioactive in mediating tau internalization into cells, and require their heparan sulfate chains.

During development, GPC4 is proteolytically shed from the cell surface of astrocytes and, once released into the extracellular space, modulates the maturation of excitatory synapses [44, 45]. We next asked whether Alzheimer’s disease pathology can lead to GPC4 shedding from microglia thereby releasing soluble GPC4 into the extracellular space. To measure GPC4 shedding in iTF Microglia, we constructed a luminescence reporter system by genetically fusing NLuc after the signal peptide of GPC4 (NLuc-GPC4) as has been previously reported [44]. Luminescence can then be measured in the iTF Microglia conditioned media to quantify the shedding of GPC4 in various disease-relevant conditions (Fig. 6B). We treated Nluc-GPC4 expressing iTF Microglia with Aβ_40_ or Aβ_42_ fibrils for 24 h and observed a dose-dependent increase, up to 10-fold, in shed GPC4 as measured by luminescence in the conditioned media (Fig. 6C). To address whether increased GPC4 shedding results from enhanced proteolysis or simply reflects increased total GPC4 production, we normalized shedding to total cellular NLuc-GPC4 by additionally measuring luminescence from the cell lysates. The proportion of GPC4 that was shed (~ 80%) remained constant across Aβ treatments, suggesting that Aβ-induced increases in shed GPC4 is primarily driven by increased GPC4 production, not increased shedding efficiency (Fig. S9B). GPC4 shedding was not observed with scrambled Aβ nor when the experiment was performed in the presence of sodium azide, a metabolic inhibitor of ATP production, suggesting that this phenomenon is amyloid-specific and requires cell metabolism (Fig. S9C and D). To further interrogate the mechanism of shedding, we tested Marimastat, a broad-spectrum MMP inhibitor. Marimastat significantly reduced Aβ-induced GPC4 shedding from 80 to 20%, supporting a role for proteolytic cleavage (Fig. S9E).

Given the possibility that Aβ aggregates can result in the shedding of GPC4, we next asked if soluble GPC4 is sufficient to promote tau phagocytosis into iTF Microglia. We co-applied pHrodo-labeled tau fibrils with increasing doses of recombinant, soluble GPC4 lacking a GPI-anchor. We observed that soluble GPC4 was sufficient to dose-dependently augment tau phagocytosis (Fig. 6D), consistent with the hypothesis that extracellular GPC4 may potentiate tau aggregate internalization in microglia.

APOE is known to potentiate tau pathology and increase tau aggregate internalization into neurons [22, 46, 47], as well as interact with astrocyte-secreted GPC4 to drive tau hyperphosphorylation [48]. Since APOE was significantly enriched in our surfaceomics dataset for microglia treated with Aβ fibrils (Fig. 1A and B), we next investigated whether APOE can alter microglial phagocytosis of tau aggregates. We confirmed that microglia secrete APOE after stimulation with Aβ_40_ or Aβ_42_ fibrils using an APOE ELISA on conditioned media (Fig. S9F). Further, Aβ_40_ and Aβ_42_ fibrils upregulated APOE transcripts as measured by qPCR, suggesting that the increase abundance of APOE in the conditioned media reflects both transcriptional induction and enhanced APOE secretion (Fig. S9G).

We then asked whether soluble APOE is sufficient to potentiate tau aggregate phagocytosis in microglia. We co-applied mammalian-derived, lipidated APOE3 [49], the most common allele in human populations, with tau-pHrodo fibrils. We observed a significant and dose-dependent increase in tau fibril phagocytosis with the addition of APOE3 (Fig. 6E). Lastly, we asked whether the combined addition of GPC4 and APOE3 could further potentiate tau phagocytosis. We applied tau-pHrodo fibrils with either GPC4 alone, APOE3 alone, or both GPC4 and APOE3. We observed that the addition of GPC4 and APOE3 together significantly potentiated tau aggregate phagocytosis relative to treatment with GPC4 or APOE3 alone (Fig. 6F). The addition of N-terminal APOE3 lacking the lipid-binding C-terminal domain, did not result in any augmentation of tau phagocytosis (Fig. S9H). Taken together, these data suggest that GPC4 and APOE3 work in concert to drive tau aggregate phagocytosis in microglia.

### Tau binds to GPC4 on the cell surface

We next investigated whether GPC4 mediates tau binding to the cell surface. We expressed either GPC4 WT or heparan sulfate deficient GPC4-ΔHS in HEK cells and measured their ability to facilitate cell surface binding of tau-647 fibrils at 4 °C. GPC4 WT expression resulted in ~ 3.5-fold more tau fibril binding to the cell surface relative to the NLuc expression control vector (Fig. S10 A) confirming that GPC4 is sufficient to mediate cell surface binding of tau fibrils. In contrast, GPC4-ΔHS minimally augmented tau cell surface binding, further corroborating the importance of the heparan sulfate chains in facilitating interactions with tau. Additionally, when HEK293T cells were transfected with a secreted form of NLuc-GPC4 WT, we successfully pulled down NLuc-GPC4 from the conditioned media using monomeric tau-biotin immobilized on streptavidin beads, as measured by luminescence (Fig. S10B). This interaction was significantly enhanced by the addition of APOE3, suggesting that tau, GPC4, and APOE3 may form a multiprotein complex.

However, we were unable to detect direct biophysical interactions between tau, GPC4, and APOE3 when using recombinant purified proteins in solution. Biolayer interferometry (Fig. S10C), protein pull-down assays (Fig. S10D and S10E), and lysine crosslinking with disuccinimidyl sulfoxide (DSSO) (Fig. S10F) did not reveal direct protein-protein interactions. These data indicate that the cellular expression of GPC4 WT is sufficient to mediate cell surface binding of tau, but that purified recombinant GPC4 is insufficient to bind tau in solution. This suggests the involvement of an additional, yet unidentified, co-factor or cellular process in mediating interactions between tau, GPC4 and APOE3.

### Microglia-derived GPC4 and APOE interact to amplify tau seeding in iPSC neurons

According to the prion hypothesis of Alzheimer’s disease, misfolded tau can propagate from one neuron to another, spreading tau pathology across the brain. The process involves the release of misfolded tau from a donor cell, uptake by a recipient cell, and subsequent templating where the misfolded tau induces normal tau protein to misfold and aggregate [50, 51]. Our data demonstrate that microglia release GPC4 and APOE in response to Aβ fibrils which, in turn, potentiates tau internalization. Given that soluble factors can act *in trans* and not only *in cis*, we asked whether soluble GPC4 and APOE can augment tau fibril uptake and pathological seeding in neurons.

**Fig. 7:**
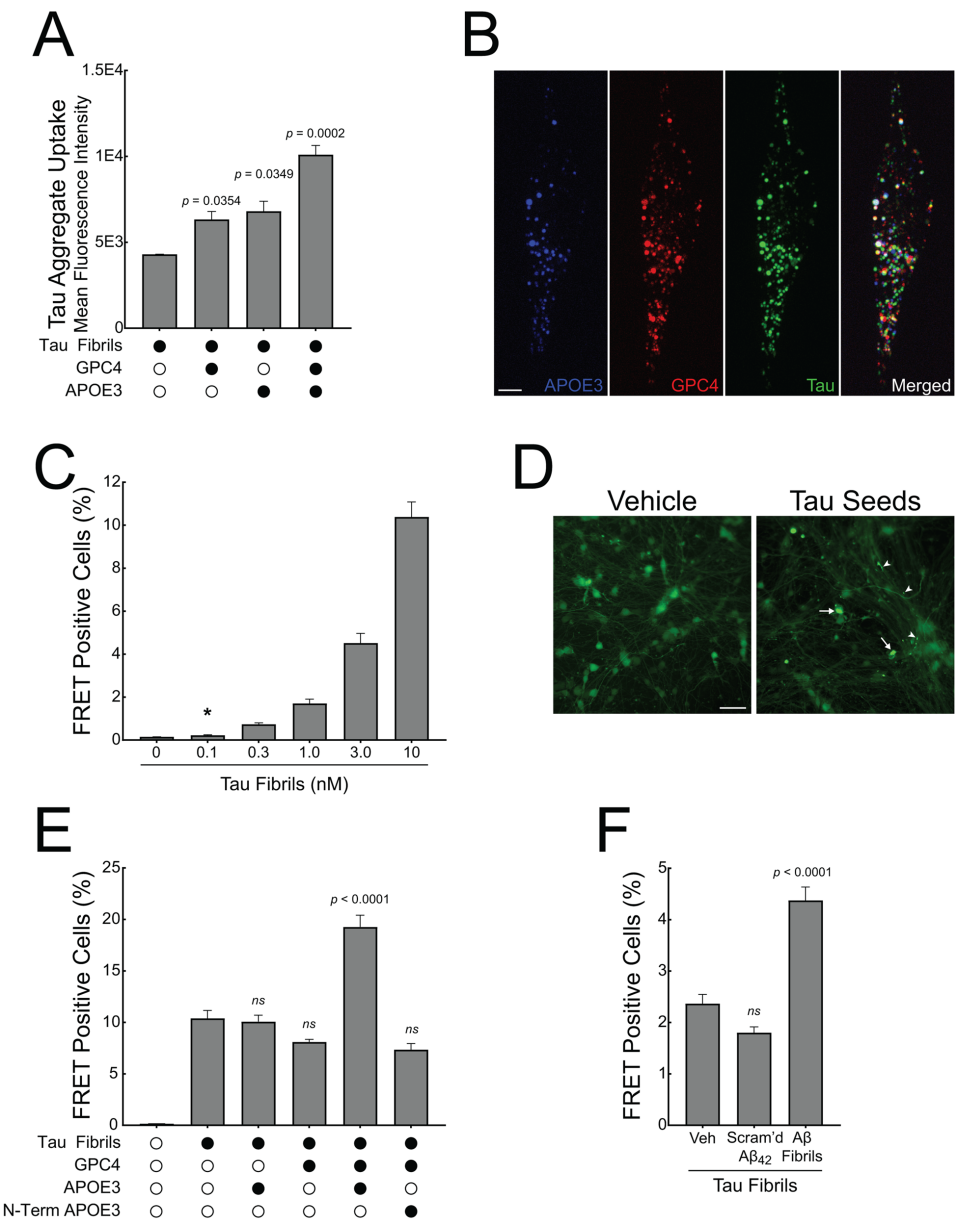
GPC4 and APOE3 amplify tau seeding in iNeuron FRET biosensors. (**A**) Uptake of tau-pHrodo red fibrils (50 nM) with or without GPC4 or APOE3 (250 nM) measured at 16 h in SH-SY5Y cells. (**B**) SH-SY5Y cells were co-incubated with tau-647 fibrils, GPC4-546 or APOE3-488 for 16 h, trypsinized, replated, and then imaged on a confocal microscope 4 h later. Scale bar = 10 μm. (**C**) iNeuron tau FRET biosensors were treated with varying doses of full-length tau fibrils for 7 days prior to measuring intraneuronal tau pathology by FRET flow cytometry. The assay is linear and statistical significance is first reached at a tau fibril concentration of 0.1 nM. (**D**) Confocal images of iNeuron tau FRET biosensors treated with vehicle or 10 nM tau fibrils for 7 days. The arrows mark tau aggregates within the neuron soma and the arrowheads mark tau aggregates within neuron processes. Scale bar = 40 µm. (**E**) iNeuron tau FRET biosensors were treated with tau fibrils (10 nM) with or without GPC4, APOE3, or N-terminal APOE3 (50 nM) for seven days prior to FRET flow cytometry. (**F**) iNeuron tau FRET biosensors were treated with Aβ_40/42_-primed microglia conditioned media containing 10 nM tau fibrils for 7 days prior to FRET flow cytometry. The statistical analyses were performed with a one-way ANOVA and Holm-Sidak multiple comparisons test. *N* = 4. The data represent the means ± SEM.

We co-applied tau-647 fibrils to SH-SY5Y cells, a human neuroblastoma cell line, along with GPC4 and APOE3, either alone or in combination, and measured internalization via flow cytometry 16 h later. We found that GPC4 and APOE3 alone increased tau aggregate internalization by 1.5-fold and 1.6-fold, respectively, while their co-application increased tau aggregate uptake by 2.35-fold (Fig. 7A). These findings are consistent with our previous results showing that GPC4 and APOE3 together enhance tau aggregate phagocytosis in iTF Microglia (Fig. 6F). To evaluate whether tau fibrils, GPC4, and APOE3 colocalize within treated cells, we performed confocal microscopy on living SH-SY5Y cells. Tau-647 fibrils were co-treated with GPC4-546 and APOE3-488 for 16 h, followed by trypsinization to remove cell surface bound protein, replating, and confocal imaging. We observed colocalization of tau with both GPC4 and APOE3 (Fig. 7B and S11A), suggesting that these proteins are localized within the same cellular compartments after internalization. To quantitatively assess the degree and extent of this colocalization, we generated colocalization scatter plots (fluorocytograms) and performed pixel-wise intensity correlation (Pearson’s r) and spatial co-occurrence analysis (Manders’ coefficients). These analyses revealed moderate intensity correlation between tau and both APOE3 (*r* = 0.7413 ± 0.006) and GPC4 (*r* = 0.7433 ± 0.007), along with partial spatial overlap (Manders M1 = 0.639 and 0.564, respectively), indicating that these internalized proteins frequently—but not completely—reside within shared intracellular compartments (Fig. S11B and C).

Next, we asked whether the co-application of GPC4 and APOE, along with tau fibrils, could potentiate tau aggregation in a neuronal culture system. Previously, we generated a HEK293T tau FRET biosensor cell line that specifically detects tau seeds in the femtomolar range [52, 53]. Here, we report the development of a more physiologically relevant biosensor based on iPSC neurons stably expressing the tau repeat domain (RD) with the disease-associated P301S mutation fused to either mClover or mApple. At baseline, the biosensor reporter proteins are monomeric. However, the application of exogenous tau seeds induces tau reporter protein aggregation, resulting in a FRET signal that can be measured with microscopy or flow cytometry. To validate this system, we applied a dose range of unlabeled, recombinant 2N4R tau fibrils to iNeuron biosensor cells without the addition of liposomes. After 7 days, the tau seeds induced a dose-dependent increase in intraneuronal tau pathology, with 100 pM of exogenous tau seeds initiating tau aggregation, demonstrating that the iNeuron biosensor system is sensitive and quantitative (Fig. 7C and D).

We then applied tau seeds (10 nM) along with recombinant GPC4 and APOE3 (50 nM) and measured tau pathology after 7 days. Tau seeds alone resulted in FRET positivity in approximately 10% of the iNeuron biosensors. This response was not augmented by the addition of either GPC4 or APOE3 alone. However, the co-application of tau seeds with both GPC4 and APOE3 resulted in a doubling of FRET positive neurons (Fig. 7E). These data suggest that GPC4 and APOE3 work together to augment tau seeding in iNeurons.

Finally, we primed iTF Microglia with 1 µm Aβ_40/42_ fibrils for 24 h to induce secretion of GPC4 and APOE3, then harvested the conditioned medium. We mixed the conditioned media with 10 nM tau fibrils and applied the mixture to the iNeuron biosensors for 7 days. Aβ-primed microglia conditioned media significantly potentiated tau seeding compared to vehicle-primed or scrambled Aβ_42_-primed cells (Fig. 7F), confirming that Aβ fibrils induce microglial secretion of factors that enhance tau pathology in neurons.

### Peri-Plaque GPC4 expression correlates with neuritic tau pathology in human Alzheimer’s disease brain

**Fig. 8:**
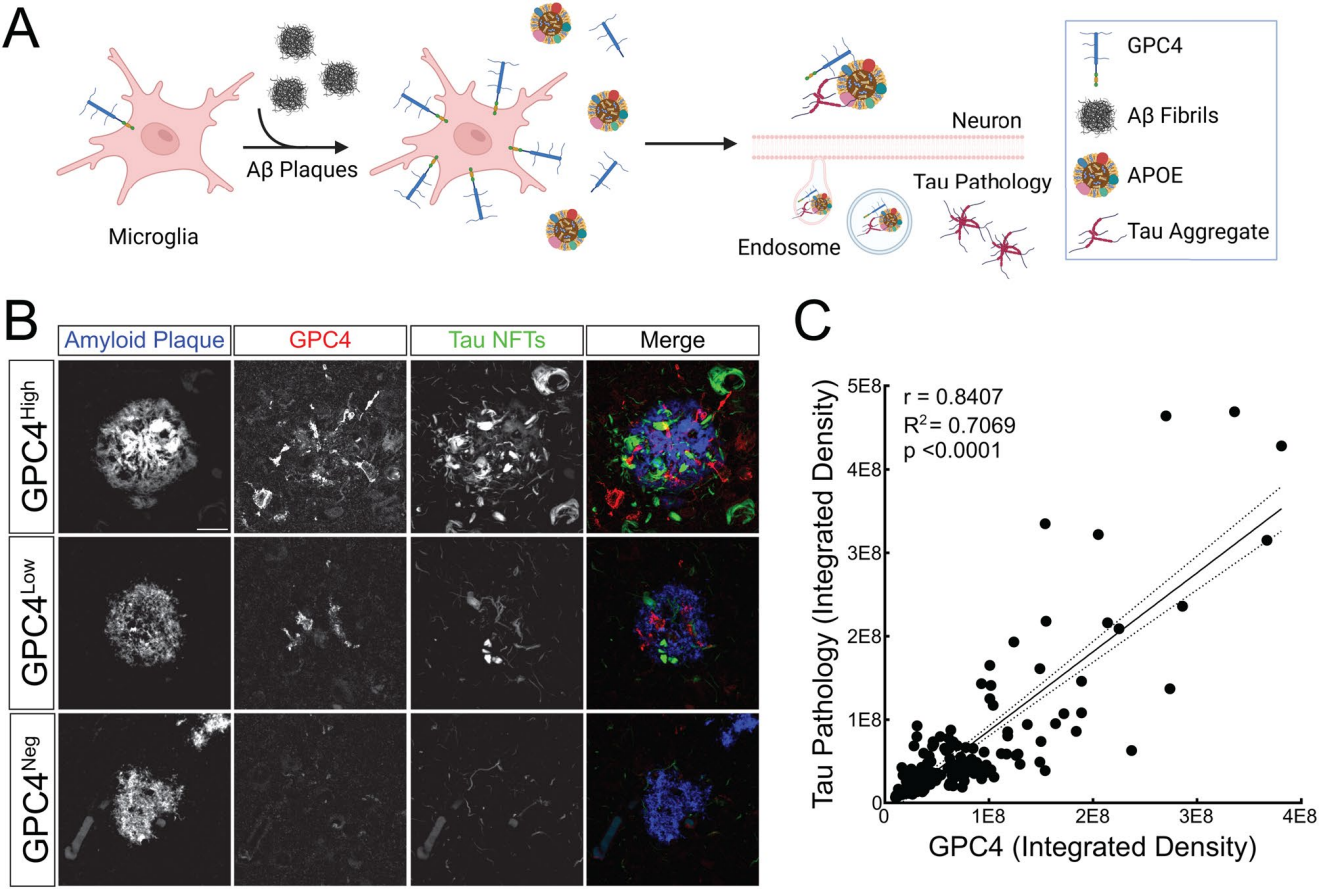
Peri-plaque GPC4 correlates with neuritic tau pathology. (**A**) Aβ fibrils stimulate microglia to upregulate GPC4 on their cell surface. In response to Aβ, GPC4 is proteolytically shed into the extracellular space, releasing a soluble proteoform. Concurrently, Aβ-exposed microglia secrete APOE, which interacts with GPC4 and tau to form a tripartite complex. This complex potentiates tau neuronal uptake and pathological seeding, ultimately contributing to the spread of tau pathology. (**B**) Representative confocal images of GPC4 (red) and amyloid plaques (blue) and tau pathology (green) using the BF-188 dye in AD brain tissue. Three representative plaques with varying degrees of GPC4 are depicted. Scale bar = 20 μm. (**C**) Scatter plot showing a positive correlation between GPC4 and tau pathology within individual amyloid plaques among four AD cases. *N* = 215 plaques. The *p*-value was determined by Pearson’s correlation with 95% confidence bands.

We propose a working model in which Aβ fibrils induce microglial expression of GPC4, a cell surface and soluble HSPG that, along with APOE, enhances tau uptake and seeding in nearby neurons (Fig. 8A). Although GPC4 expression does not directly correlate with global tau pathology in AD or primary tauopathy patients (Fig. 3E), we asked whether GPC4 expression may locally correlate with neuritic plaques. Since neuritic plaques are defined by the presence of dystrophic tau-positive neurites surrounding a central Aβ core, whereas compact plaques lack associated tau pathology, we hypothesized that GPC4 expression would correlate with the burden of tau pathology residing within amyloid plaques. To perform a plaque-level analysis, we stained human AD brains with GPC4 antibodies and BF-188, a conformation-sensitive fluorescent dye that binds to β-sheet–rich protein aggregates. BF-188 has distinct spectral signatures for amyloid and tau, enabling simultaneous visualization and spectral deconvolution of these two pathological proteins within the same tissue section.

We analyzed 215 amyloid plaques across four AD brains. Representative high-magnification images showed distinct GPC4 signal within and around amyloid plaques and adjacent tau-positive neurites (Fig. 8B and Supplementary Material 15). Quantitative analysis across plaques demonstrated a robust, statistically significant correlation between GPC4 and local tau pathology (*r* = 0.84, R² = 0.71, *p* < 0.0001) (Fig. 8C). To assess whether this association was consistent across individuals, we performed the same spatial quantification in the four AD cases separately. In each case, peri-plaque GPC4 intensity was significantly correlated with neuritic tau pathology (Fig. S12). Correlation coefficients ranged from *r* = 0.76 to *r* = 0.88, with each case reaching statistical significance (*p* < 0.0001). Together, these findings support a consistent and reproducible association between GPC4 expression and the local aggregation of tau within neuritic plaques in human AD brain.

## Discussion

Aβ and tau are critical proteins in the pathogenesis of AD, but the mechanisms by which they interact to promote disease remain unclear. Our study delineates a role for microglia in mediating the interaction between Aβ and tau pathology through the expression of the GPI-anchored heparan sulfate proteoglycan, GPC4. In concert with APOE3, GPC4 enhances the entry of tau seeds into both microglia and neurons, with neuronal entry facilitating subsequent tau pathology.

To catalogue the human iPSC microglia surfaceome in response to Aβ, we first employed a rapid and sensitive cell surface proteomic strategy using WGA-HRP [27]. Our proteomics data revealed that Aβ-exposed microglia upregulate heparan sulfate proteoglycans and the heparan sulfate-binding protein, APOE. These changes occurred within a broader remodeling program enriched for pathways related to proteoglycan processing, extracellular matrix reorganization, lipid metabolism, and endocytosis. Using flow cytometry, we validated that microglia selectively upregulate heparan sulfate and GPC4 when treated with amyloids but not cognate monomer or innate immune activators. Additionally, iPSC astrocytes, iPSC neurons, and immortalized cell lines minimally altered their cell surface heparan sulfate in response to Aβ fibrils, likely due to their limited capacity to internalize fibrillar Aβ. Thus, the process of Aβ-induced microglia priming may exhibit both substrate and cell-type specificity. Our observation that Aβ fibrils induce robust surface GPC4 expression in microglia is also consistent with recent large-scale proteomic analyses of human AD brain tissue. For example, Bai et al. reported elevated GPC4 levels in bulk human AD brain lysates [54]. While these datasets are not microglia-specific, their concordance with our targeted microglia models provides independent support for the relevance of GPC4 upregulation in AD.

We validated these findings in human AD post-mortem tissue. Specifically, we found that microglial GPC4 expression was enriched around Aβ plaques, and that overall microglial GPC4 levels correlated with plaque burden, but not with global neurofibrillary tangle load. However, when we stratified plaques by GPC4 intensity, we found a strong correlation with local neuritic tau pathology, suggesting that microglial GPC4 may influence the regional development of tau aggregates and, by extension, neuritic plaques.

Our study does not refute the possibility that other CNS cell types may express GPC4. Indeed, a recent report found that astrocyte-secreted GPC4 preferentially interacts with APOE4 to promote tau hyperphosphorylation [48]. Notably, in that study, GPC4 levels were modestly elevated (~ 0.25-fold) in AD patients homozygous for APOE4, and remained unchanged in APOE3/3 and APOE2/2 patients compared to age-matched controls. This suggests that astrocyte GPC4 expression may be driven primarily by APOE genotype, rather than Aβ pathology.

In contrast, our data show that AD-associated microglia upregulate GPC4 by 2.2-fold in regions of Aβ plaque deposition. Furthermore, three of four subjects in our cohort were APOE3/3, and one was APOE3/4 (Supplementary Material 14), indicating that microglial GPC4 upregulation occurs independently of APOE4 homozygosity. Consistent with this finding, the iTF Microglia used in our cell culture experiments are derived from an APOE3/3 iPSC line [55], further supporting the conclusion that GPC4 upregulation is driven by Aβ exposure rather than APOE genotype. Thus, microglia appear to be the primary cell type responding to Aβ by increasing GPC4 expression. To our knowledge, this is the first report linking any glypican family member to microglia biology. These data raise the possibility that a complex interplay between glial GPC4, APOE isoform, and Aβ burden may modulate tau pathology in AD.

To investigate whether GPC4 modulates Aβ-associated deficits in vivo, we used a *Drosophila* amyloidosis model that expresses human Aβ_42_ pan-neuronally [41]. This model displays decreased climbing activity and early lethality. We found that glial-specific over-expression of *Dlp*, the *Drosophila* ortholog of GPC4, exacerbated Aβ-mediated toxicity, whereas the knockdown of glial *Dlp* rescued Aβ-mediated toxicity. Importantly, these phenotypes were not recapitulated when *Dlp* expression was modulated in control animals, suggesting that the impact of GPC4 is dependent on the presence of Aβ pathology. While we cannot exclude the contribution of endogenous *Drosophila* tau (dTau) as a modifier of Aβ neurotoxicity, there is no evidence that dTau spontaneously aggregates or mediates toxicity in this model, and Aβ-induced phenotypes in flies have been shown to occur independently of tau [56, 57]. We also note that neither the *Drosophila* nor the murine models of amyloidosis used in this study show elevated glial GPC4/*Dlp* expression, highlighting a limitation of these in vivo systems for modeling innate Aβ-induced microglial responses. A recent proteomic study by Yarbro et al. found that GPC4 is not upregulated in microglia from 5xFAD, NLF, and NLGF mice [58]. This result is concordant with our findings and may reflect the broader evolutionary divergence of microglial responses, with human microglia exhibiting unique transcriptional programs not conserved in rodents or flies. Nevertheless, the genetic accessibility of Drosophila glia enabled us to directly manipulate *Dlp* in vivo and test its role in modulating Aβ toxicity. These findings support a functional interaction between Aβ and glial GPC4 in vivo, though the molecular mechanisms mediating these phenotypes remain unclear.

Our prior work establishes that cell surface HSPGs mediate the internalization of proteopathic amyloids including tau and synuclein, facilitating the prion-like spread of pathology from cell to cell [35–37, 59, 60]. We therefore reasoned that GPC4 may similarly mediate the uptake of tau aggregates. Indeed, other groups have demonstrated that GPC4 enables the internalization of Aβ in neural stem cells [61], tau oligomers in astrocytes [62], or tau monomer in neurons [48]. Here, we found that Aβ primes microglia to express cell surface heparan sulfate and GPC4. Using a combination of pharmacologic-, genetic-, and antibody-based inhibition, we found that GPC4 regulates the binding and phagocytosis of tau aggregates into microglia, consistent with the established role of HSPGs as a receptor for tau aggregates. Unexpectedly, however, we also found that Aβ leads to GPC4 cell surface shedding, resulting in soluble, extracellular GPC4 proteoforms. This shedding was partially inhibited by Marimastat, a broad-spectrum MMP inhibitor, suggesting that proteolytic cleavage may contribute to the release of GPC4 into the extracellular space. Soluble GPC4 retained bioactivity in facilitating tau phagocytosis suggesting that the cell surface anchored proteoform of GPC4 may not be the only pathogenic form. The specific MMPs involved, and the precise cleavage sites, remain to be determined. Interestingly, a recent clinical study found that elevated GPC4 levels in the CSF and serum of Parkinson’s disease patients correlated with worse cognitive function [63], raising the possibility that soluble GPC4 may also be involved in synucleinopathies. Alternatively, this association could reflect comorbid Aβ pathology, which is common in Parkinson’s disease with dementia and may similarly trigger microglial GPC4 release.

Classically, GPC4 is an established effector of CNS development. For example, astrocyte-derived GPC4 and GPC6 induce the formation of functional synapses in developing mouse retinal ganglion neurons [45]. In rats, GPC4 is localized to neuronal pre-synaptic membranes where it regulates the development of excitatory neurons [64]. In the developing mouse cerebral cortex, GPC4 is expressed in neural precursor cells to regulate FGF2 signaling and cortical neurogenesis [65]. Here, we propose a role for GPC4 in microglia biology in the context of neurodegeneration. As GPI-anchored proteins, glypicans possess the unique ability to detach and reinsert themselves into the plasma membrane, enabling lateral cell-to-cell translocation and ligand gradient formation, a process referred to as *hopping* [66]. During development, hopping is exploited to generate morphogen gradients for the proper patterning of CNS topology. For example, in *Drosophila*, the extracellular distribution of secreted Wnts is established via the GPC4 ortholog, *Dlp*, which facilitates short- and long-range signaling gradients to ensure proper formation of the germarium and wing discs. Further, *Dlp* shields Wnt’s lipid moiety from the aqueous environment effectively solubilizing the ligand and permitting its spread to more distant sites [67]. It is intriguing to speculate that, in neurodegeneration, GPC4 hopping may be hijacked to traffic pathologic proteins, such as tau or inflammatory cytokines, from amyloid plaques to distant sites via diffusion-based gradients as has been observed for synthetic GPI-anchored ligands [66]. Since the heparan sulfate chains are required for GPC4’s tau-potentiating activity, the cleavage site must lie C-terminal to the glycosaminoglycan attachment sites. We found that soluble GPC4 retains its ability to facilitate tau phagocytosis, implying that these modifications are preserved. Indeed, a recent study demonstrated that astrocyte-derived GPC4 is shed with its heparan sulfate chains intact [44], supporting this interpretation. Future studies should test whether this mechanism can explain the network diffusion models of disease spread in neurodegeneration [7, 68, 69].

Coincident with this hypothesis, we observed that the lipid-bearing protein, APOE is also secreted by Aβ - treated microglia. APOE is a heparan sulfate-binding protein [70], and APOE3 and APOE4 facilitate tau aggregate entry into neurons, whereas the protective R136S Christchurch mutation abrogates tau uptake [47, 71]. Astrocytes are the primary cell type to express APOE in the healthy human brain. However, in AD, astrocytes downregulate and microglia upregulate APOE production [72–74]. We therefore hypothesized that microglia-secreted GPC4 and APOE may work together to facilitate tau internalization and pathological seeding. We found that APOE3 is sufficient to stimulate tau phagocytosis in microglia, and the combination of GPC4 plus APOE3 resulted in synergistic tau uptake. Although phagocytosis of tau aggregates in microglia could be a neuroprotective function that eliminates pathologic tau aggregates, numerous studies now suggest that microglial tau phagocytosis can instead accelerate the spread of tau pathology via extracellular vesicles [19, 75–77]. We have yet to explore the impact of GPC4 on the formation of extracellular vesicles.

To test whether soluble GPC4 and APOE cooperatively promote tau seeding in neurons, we developed a novel iPSC neuron tau FRET biosensor line. Co-application of GPC4 and APOE3 significantly enhanced pathological tau seeding, whereas either protein alone was insufficient to elicit this effect, indicating a synergistic interaction. Furthermore, conditioned media from Aβ-treated microglia also potentiated tau seeding, supporting a model in which microglia-secreted factors —including GPC4 and APOE—contribute to tau pathology.

Our data demonstrate a biological interaction between Aβ, GPC4, APOE3, and tau. However, we have yet to define the upstream mechanisms by which Aβ induces GPC4 expression. Additionally, we have not yet explored how other APOE alleles influence the interaction with GPC4. Given that APOE allele variants differ in their affinity for heparan sulfate [78, 79], it is likely that these differences modulate the capacity of APOE to cooperate with GPC4 in promoting tau pathology. Particularly intriguing is the APOE R136S Christchurch mutation, which reduces heparan sulfate binding and is associated with resilience to tauopathy [80]. We hypothesize that APOE R136S may fail to engage with GPC4, thereby disrupting the formation of a neurotoxic APOE–GPC4–tau complex. Further, in our in vivo studies we used a *Drosophila* model to investigate the role of glial GPC4 (*Dlp*); however, our genetic manipulations do not distinguish between astrocytes and cortex glia, limiting our ability to make claims about cell-type specificity to the observed effects. Moreover, since *Drosophila* tau does not spontaneously aggregate, it is unlikely that glial GPC4 exacerbates Aβ toxicity via tau pathology-related mechanisms in this model. This suggests the existence of additional, tau-independent pathways through which GPC4/*Dlp* may modulate amyloid-associated toxicity.

## Conclusions

Our study demonstrates that Aβ pathology drives the upregulation of microglial GPC4 in Alzheimer’s disease which, in conjunction with APOE, amplifies tau pathology. Through surfaceomic profiling, we identified GPC4 as a previously unrecognized microglial response to Aβ fibrils. In vivo, glial GPC4 (*Dlp*) expression exacerbates Aβ toxicity, reducing locomotion and lifespan in a Drosophila model. Microglial GPC4 mediates the phagocytosis of tau aggregates, but GPC4 can also be shed to work *in trans*. Shed GPC4 synergizes with APOE to enhance the internalization and seeding of tau aggregates into neurons. We therefore propose that microglia are particularly well-suited to respond to Aβ via a toxic GPC4 / APOE secretion axis which may accelerate neurodegeneration by amplifying tau pathology and spread in neurons.

Our findings contribute to a growing body of work implicating microglia as key orchestrators of tau propagation in Alzheimer’s disease. By identifying GPC4 as an Aβ-induced, microglia-derived factor that synergizes with APOE to enhance tau seeding, our study provides a mechanistic explanation for how microglial activation creates a permissive environment for tau pathology. This model aligns with the broader framework of microglial involvement in AD progression, particularly at the intersection of Aβ and tau pathology. For example, Pascoal et al. used in vivo PET imaging to show that regions of elevated microglial activation at baseline predicted the subsequent spread of tau pathology in a Braak-like pattern [24]. In other words, tau followed microglia, which in turn follow amyloid. Our findings suggest a molecular rationale for this observation: microglial responses to Aβ lead to the secretion of factors like GPC4, which may condition the local environment to support tau seeding and spread. This hypothesis also offers insight into the tempo of disease progression. Individuals with more robust microglial responses to amyloid—such as APOE4 carriers or those in pro-inflammatory states—may experience faster tau spread and more rapid cognitive decline, a pattern supported by several epidemiological studies [81, 82]. Conversely, a muted or altered microglial response, whether due to genetic factors or anti-inflammatory interventions, could slow the spread of tau despite ongoing amyloid deposition. Together, these observations are important for the development of therapeutic agents that target the intersection of Aβ and tau pathology.

## Methods

### Human iPSC-derived iTF Microglia cell culture and differentiation

We generated iPSC-derived iTF Microglia using a protocol established by Dräger et al., 2022 [32]. Human APOE3 homozygous iPSCs (WTC11, Coriell Cat. No. GM25256) with stably integrated doxycycline-inducible transcription factors (CEBPa, CEBPb, IRF5, IRF8, MAFB, PU1) were maintained according to established protocols [32, 55]. For differentiation, day 0 iPSCs were seeded onto plates coated with Matrigel and poly-d-lysine in Essential 8 Medium (Gibco; A1517001) supplemented with 10 µM ROCK inhibitor and 2 µg/mL doxycycline (Clontech; 631311). On day 2, the media was changed to Advanced DMEM/F12 Medium (Gibco; 12634-010) supplemented with 1 × Antibiotic-Antimycotic (Anti-Anti) (Gibco; 15240-062), 1 × GlutaMAX (Gibco; 35050-061), 2 µg/mL doxycycline, 100 ng/mL Human IL-34 (Peprotech; 200−34) and 10 ng/mL Human GM-CSF (Peprotech; 300-03). On day 4, 6, and 8 the media was replaced with Advanced DMEM/F12 supplemented with 1 × Anti-Anti, 1 × GlutaMAX, 2 µg/mL doxycycline, 100 ng/mL Human IL-34, 10 ng/mL, Human GM-CSF, 50 ng/mL Human M-CSF (Peprotech; 300−25), and 50 ng/mL Human TGF-β1 (Peprotech; 100–21C). On day 8, the differentiated iTF Microglia were used for experimentation. For CRISPRi and CRISPRa experiments, iTF iPSCs harboring trimethoprim-inducible CRISPRi or CRISPRa machinery [32] were used and maintained in 50 nM trimethoprim (MP Biomedical; 195527) starting on day 0 and continuing through the remainder of differentiation.

### Human iPSC neuron cell culture, differentiation, and generation of FRET biosensors

Pre-differentiation and differentiation of excitatory glutamatergic neurons was performed as previously described [83]. Briefly, iPSCs were split into matrigel coated plates in pre-differentiation medium (KnockOut DMEM/F12 with 1× MEM non-essential amino acids, 1× N2 Supplement [Gibco/Thermo Fisher Scientific, 17502-048], 10 ng/mL of BDNF [PeproTech, 450-02], 10 ng/mL of NT-3 [PeproTech, 450-03], 1 µg/mL of mouse laminin [Thermo Fisher Scientific, 23017-015], and 2 µg/mL of doxycycline [Takara; 631311]). 10 nM ROCK inhibitor was added on the initial passage day (Day − 3) and omitted thereafter. After a total of three days in pre-differentiation media (day 0), cells were re-plated on poly-D-lysine coated BioCoat plates (Corning) in BrainPhys media containing 0.5× N2 supplement, 0.5× B27-VA supplement, 10 ng/mL NT-3, 10 ng/mL BDNF, 1 µg/mL mouse laminin, and 2 µg/mL doxycycline. On day 3, media was replaced with complete BrainPhys media without doxycycline. Subsequently, half media changes were performed weekly unless stated otherwise. For uptake experiment, iNeurons were treated on day 7. For seeding experiments, iNeurons were treated on day 3 and harvested for flow cytometry on day 10.

To produce tau FRET biosensor iPSCs, lentivirus encoding tauRD (244–368)-mClover and mApple harboring the P301S mutation was transduced into the previously established CRISPRi-i3N human iPSC line [83] (Coriell GM29371). A polyclonal population of dual-positive TauRD(P301S)-Clover/Apple biosensors were sorted using a BD FACSAria Fusion and expanded for further use.

### Human iPSC astrocytes

Differentiation of iAstrocytes was performed as previously described [84].

### Cell culture, transfection, and viral infection of cells

HEK293 cells (ATCC, CRL-1573) were cultured in high-glucose DMEM with 10% FBS and penicillin (100 U/ml)-streptomycin (100 µg/ml). SH-SY5Y (ATCC, CRL-2266) cells were cultured in advanced DMEM/F12 (Gibco) supplemented with 10% FBS, penicillin (100 U/ml), and streptomycin (100 µg/ml). Cells were incubated in a humidified atmosphere of 5% CO_2_ at 37 °C. HEK293 cells were transfected with plasmids using Lipofectamine 3000 (Thermo Fisher Scientific; L3000008) or transduced with lentivirus encoding GPC4 WT, GPC4-secreted, GPC4-ΔHS, GPC4-secreted-ΔHS, NLuc-GPC4 WT, or NLuc-GPC4-secreted. All GPC4 DNA sequences were synthesized by Twist Biosciences and cloned into FM5 vector using a Gibson assembly method.

### Protein acquisition, expression, purification, fibrillization, and labeling

Recombinant Aβ_40_ (A-1001-2), Aβ_42_ (A-1002-2), scrambled Aβ_42_ (A-1004-1), and α-synuclein (S-1001-1) were purchased from rPeptide. Aβ_40_ and Aβ_42_ peptides were dissolved at 5 mg/mL in hexafluoroisopropanol and evaporated into a peptide film. The peptide films were resuspended at 10 mg/mL in DMSO, vortexed, and sonicated for 5 min, then diluted to 0.2 mg/mL in 10 mM sodium phosphate buffer, pH 7.4. The solution was shaken at 900 rpm at 37 °C for 72 h (Aβ_40_) or 120 h (Aβ_42_) to form fibrils. Scrambled Aβ_42_ (A-1004-1) was reconstituted at 0.2 mg/mL in PBS and stored at -80 C° until further use. α-Synuclein was fibrillized by dissolving the lyophilized protein at 2 mg/mL in PBS at 900 rpm at 37 °C for 72 h; α-Synuclein monomer was maintained by immediately storing the freshly reconstituted protein at -80 °C without shaking. Full length 2N4R tau with a C-terminal polyhistidine tag in the pet28b plasmid was expressed and prepared as previously described [85] from BL21-Gold (DE3) competent cells. Tau was purified on a Ni-NTA column and eluted in 1× PBS. To induce fibrillization of tau monomer, 8 µM tau was incubated at 37 °C in 10 mM HEPES, 100 mM NaCl, and 8 µM heparin for 72 h without agitation. Tau and magnetic beads (Spherotech; AM-10-10) were conjugated to pHrodo-red dye or AlexaFluor 647 (ThermoFisher; P36600 and A20006) by buffer exchanging the substrates into 100 mM sodium bicarbonate buffer, adding pHrodo red dye at a final molar ratio of 5:1 (dye: substrate), and incubating at RT for 15 min. Excess dye was quenched by adding glycine at a final concentration of 100 mM for 15 min and then buffer exchanging via dialysis into PBS. For protein pull-down assays, full-length tau-biotin was purchased from rPeptide (T-1114-1). Mammalian-derived and heparan-sulfated GPC4-WT was purchased from Acro (GP4-H52H3).

For APOE expression and purification, human his-tagged full-length APOE3 and N-terminal APOE3 (1–216) DNA sequences were generated by Twist Bioscience and cloned into pcDNA3.4 using a Gibson assembly method. Transient transfection of plasmid DNA was done in Expi293F cells at 37 °C. Cells were harvested on day 7 by centrifugation at 4000 x g for 20 min and the supernatant was filtered through a 0.45 µM sterile filter. The supernatant was incubated with 1mL (per 30 mL of supernatant) Ni-NTA resin (Cytiva; 17371203) and imidazole to a final concentration of 5 mM for 1 h at 4 °C with end-over-end mixing. The resin-media mixture was loaded on a chromatography column and washed with 5 resin volumes of wash buffer (PBS pH 8.0, 5 M NaCl, 50 mM K_3_PO_4_, 10 mM imidazole) for a total of two times. APOE3 was then eluted in 1 resin volume of elution buffer (0.5 M NaCl, 50 mM K_3_PO_4_, 1 M imidazole) in a total of 3 elution fractions. The purity of each fraction was assessed by SDS-PAGE and fractions > 90% pure were pooled and buffer exchanged into PBS using a 10 kDa MWCO centrifugal filter (Amicon; UFC9010). The protein concentration was determined by bicinchoninic acid (BCA), and the protein was diluted to a final concentration of 8 µM and stored at -80 °C.

### iTF Microglia cell surface labeling

For cell surface proteomic studies, the iTF Microglia cell surface was labeled with cell-tethered WGA-HRP according to the protocol established in Kirkemo et al., 2022 [27]. In brief, cells were lifted using Versene (Gibco; 15040066) for 10 min at 37 °C. Cells were diluted in DPBS and pelleted by centrifugation at 1500 g for 5 min and resuspended in DPBS pH 6.5. Cell surfaces were labeled by incubating cells with 0.5 µM WGA-HRP for 5 min on ice, then adding 500 µM biotin tyramide, and finally adding 1 mM of H_2_O_2_. The mixture was incubated at 37 °C for 2 min. The reaction was quenched with 10 mM Sodium Ascorbate/1 mM Sodium Pyruvate followed by two additional washes in the same buffer prior to a final wash in DPBS. Cells were pelleted and flash frozen before further processing.

### Proteomic preparation for surface-labeled iTF cells

Frozen cell pellets were thawed and processed for LC-MS/MS using a preOmics iST kit (P.O. 00027). Briefly, cell pellets were lysed in 2x RIPA buffer (Millipore Sigma; 10–188) containing protease inhibitors (cOmplete, Mini, EDTA-free Protease Inhibitor Cocktail; Millipore Sigma; 11836170001) and 1.25 mM EDTA. Cells were further disrupted via sonication. Biotinylated proteins were pulled down with NeutrAvidin-coated agarose beads (ThermoScientific; 29204) for 1 h at 4 °C. Beads were transferred to Poly-Prep chromatography columns (Bio-Rad) and sequentially washed with 1x RIPA buffer, high-salt PBS (PBS pH 7.4, 2 M NaCl), and denaturing urea buffer (50 mM ammonium bicarbonate, 2 M Urea). From the PreOmics iST kit, 50 µL of the provided LYSE solution was added to the slurry and the mixture was incubated at 55 °C for 10 min with shaking. The provided enzyme mixture (Trypsin and LysC) was resuspended in 210 µL of RESUSPEND buffer, mixed, and 50 µL was added to the slurry. Samples were allowed to mix at 500 rpm for 90 min at 37 °C, before being quenched with 100 µL of STOP solution. Samples were desalted using the provided Preomics columns and wash buffers per the manufacturer’s instructions. Peptides were eluted with 2Χ 100 µL of ELUTE, dried, and resuspended with 2% acetonitrile and 0.1% TFA. Peptides were quantified using Pierce Quantitative Colorimetric Peptide Assay (Thermo Fisher Scientific, 23275).

### LC-MS/MS and data analysis

Liquid chromatography and mass spectrometry were performed as described previously [27]. Briefly, 200 ng of samples were separated over a 90-minute linear gradient of 3–35% solvent B (Solvent A: 2% acetonitrile, 0.1% formic acid, solvent B: acetonitrile, 0.1% formic acid) on either an Aurora Ultimate CSI 25 cm×75 μm C18 (Ionopticks) or a PepSep XTREME 25 cm x 150 μm (Bruker) UHPLC column using a nanoElute UHPLC system (Bruker), and injected into a timsTOF pro mass spectrometer (Bruker). Data-dependent acquisition was performed with parallel accumulation-serial fragmentation (PASEF) and trapped ion mobility spectrometry (TIMS) enabled with 10 PASEF scans per topN acquisition cycle. For database searching, peptides were searched using MaxQuant’s (Version 2.6.1) Andromeda search engine against the plasma membrane annotated human proteome (Swiss-prot GOCC database, June 3, 2020 release). Enzyme specificity was set to trypsin + LysC with up to two missed cleavages. Cysteine carbamidomethylation was set as the only fixed modification; acetylation (N-term) and methionine oxidation were set as variable modifications. The precursor mass error tolerance was set to 20 PPM and the fragment mass error tolerance was set to 0.05 Da. Data was filtered at 1% for both protein and peptide FDR and triaged by removing proteins with fewer than 2 unique peptides. GPC6 was identified via a single peptide and was thus excluded from all proteomic analyses with the exception of Fig. 1E where it was included to compare to other glypicans. All mass spectrometry database searching was based off of at least three biological replicates. Biological replicates underwent washing, labeling, and downstream LC-MS/MS preparation separately. Perseus was used to analyze label-free quantitation data generated in MaxQuant. All peak areas were log2(x) transformed and missing values were imputed separately for each sample using the standard settings (width of 0.3, downshift of 1.8). Significance was based off of a standard unpaired Student t test with unequal variances across all replicates. Reported peak area values represent the averages of all replicates. Proteins with a −/+1.5-fold change and *p* < 0.05 were included in downstream analysis, and this threshold was chosen to balance the identification of biologically relevant changes while maintaining statistical rigor [86]. For representation of the data in figures, a Z-score was computed and is defined as (LFQ Area - Mean LFQ Area)/Standard Deviation. Protein IDs that were not annotated to be secreted or expressed extracellularly were removed. Heatmaps comparing expression levels between donors were generated with heatmapper.ca using an average linkage clustering method with Euclidean distance.

### Immunocytochemistry

All iTF Microglia were grown on µ-slides (Ibidi; 80804) for imaging studies. For immunocytochemistry of microglia markers, day 8 iTF Microglia were washed with PBS solution, fixed with 4% PFA, and blocked with 10% normal goat serum in PBS. Cells were stained with anti-IBA1 rabbit primary antibody (1:50; Cell Signaling Technology), anti-PU.1 rabbit primary antibody (1:100), and anti-TMEM119 mouse primary antibody (1:100) overnight at 4 °C followed by secondary labeling with Alexa Fluor 488 anti-mouse or Alexa Fluor 546 anti-rabbit (1:2000) and Hoechst 33342 (Invitrogen; H1399) for 1 h at RT. For the intracellular antigens IBA1 and PU.1, cells were permeabilized in 0.25% Triton X-100 prior to the addition of primary antibodies. For glypican staining, cells were treated with 1 µM Aβ_40_ or Aβ_42_ fibrils for 24 h, washed three times in PBS, and fixed as described earlier. Cells were then blocked with 10% NGS and stained with GPC1 (1:100; Abcam), GPC2 (1:100; Invitrogen), GPC3 (1:200; Invitrogen), GPC4 (1:100; ProteinTech), GPC5 (1:200; R&D Systems), and GPC6 (1:100; Bioss) overnight at 4 °C followed by secondary labeling with Alexa Fluor 488 anti-mouse or Alexa Fluor 647 anti-rabbit (1:2000) and Hoechst 33,342. For Aβ fibril and GPC4 correlational analysis (Fig. S4), cells were treated with pHrodo-red labeled Aβ fibrils for 24 h, and then live cells were immunostained with a GPC4 primary antibody followed by secondary antibody for one hour each at 37 °C prior to live-cell confocal imaging. Individual iTF Microglia were segmented using the brightfield channel with FIJI’s Trainable Weka Segmentation plugin [87], and each cells’ GPC4 and Aβ-pHrodo integrated fluorescence intensity was separately quantified. To perform colocalization analysis on SH-SY5Y cells treated with fluorescently labeled tau fibrils, APOE3, and GPC4 (Fig. 7B and S11), images were acquired by confocal microscopy and analyzed using FIJI (ImageJ) with the JACoP plugin. To minimize the contribution of background signal and restrict colocalization analysis to internalized puncta, we first generated an ROI mask based on the combined intensity of all three fluorescent channels. The summed image was auto-thresholded using the Otsu method to create a binary mask defining intracellular puncta. This mask was applied to each individual channel to isolate foreground regions for colocalization analysis. Fluorocytograms, Pearson’s correlation coefficients, Manders’ overlap coefficients (M1 and M2) were generated using JACoP [88, 89]. All analyses were restricted to non-zero voxels to avoid inflation of colocalization metrics by background signal.

### Human brain tissue and immunohistochemistry

Sixteen human brain tissue samples were obtained from the Neurodegenerative Disease Brain Bank at the University of California, San Francisco (Supplementary Material 14). Prior to autopsy, patients or their surrogates provided informed consent for brain donation, in keeping with the guidelines put forth in the Declaration of Helsinki. Neuropathological diagnoses were made following consensus diagnostic criteria [90] using previously described histological and immunohistochemical methods [91, 92, 93]. Cases were selected based on neuropathological diagnosis. Healthy control tissues were obtained from individuals without dementia who had minimal age-related neurodegenerative changes. Formalin-fixed blocks of the middle frontal gyrus were cut from coronal slabs and embedded together into tissue arrays in paraffin wax. Each tissue array contained four small tissue blocks with one tissue block from each histopathologic category (amyloid-/ tau-, amyloid+/tau-, amyloid-/tau+, and amyloid+/tau+). For immunofluorescence, tissue arrays were sectioned at 8 μm using a rotary microtome. The case details are listed in Table 1.

To reduce the autofluorescence of human brain tissue, glass-mounted sections were photobleached for 72 h using a multispectral LED array in a cold room [94]. The sections were baked at 65 °C for 30 min and deparaffinized and rehydrated followed by antigen retrieval in an autoclave at 120 °C for five minutes using 10 mM citrate buffer (pH 6). The tissue sections were permeabilized in PBS containing 0.25% Triton X-100 (PBS-T) and blocked in 10% normal goat serum for 1 h. Primary antibodies were diluted in PBS-T and 10% normal goat serum and applied to the slides overnight at RT. The sections where then washed in PBS-T and secondary antibodies diluted 1:500, with or without DAPI, were applied at RT for two h. For experiments requiring amyloid plaque fluorescent staining, Amylo-Glow RTD (Biosensis; TR-300-AG) was applied for 10 min per the manufacturer’s directions after the secondary antibody application. For experiments requiring amyloid plaque and neurofibrillary tangle fluorescent staining, 5 µM BF-188 (FujiFilm; 025-18801) was applied for 30 min after the secondary antibody application. The sections were washed with PBS and coverslipped with Fluoromount-G (ThermoFisher; 00-4958-02). For quantification of amyloid plaque and neurofibrillary tangle burden, tissue sections were deparaffinzed, peroxidase-blocked with 3% H_2_O_2_ in methanol for 30 min, and, for amyloid plaque staining, pre-treated with 88% formic acid for 6 min. Antigen retrieval and antibody staining was performed as described above. DAB reactions were developed by applying Vectastain ABC Elite (Vector Laboratory; PK-6200) followed by 0.05% diaminobenzidine and counterstaining with hematoxylin. Antibodies used in this study include: anti-IBA1 guinea pig primary antibody (1:250; Synaptic Systems), anti-Glypican 1 rabbit primary antibody (1:50; Abcam), anti-Glypican 2 rabbit primary antibody (1:50; Invitrogen), anti-Glypican 4 rabbit primary antibody (1:50; ProteinTech), anti-Glypican 6 rabbit primary antibody (1:50; Bioss Antibodies), anti-Aβ mouse primary antibody (DE2; 1:500; Millipore Sigma), anti-phosphorylated tau (CP13; 1:250; Peter Davies), Alexa Fluor 488 goat anti-guinea pig secondary antibody (1:500; Invitrogen), Alexa Fluor 647 goat anti-rabbit IgG secondary antibody (1:500; Invitrogen), and biotinylated horse anti-mouse secondary antibody (1:200; Vector).

### Human brain tissue microscopy and analysis

Confocal images were generated using a Nikon Ti2-E microscope equipped with a Crest X-Light-V2 spinning disk confocal (Crest Optics), Celesta Light Engine (Lumencor), Piezo stage (Mad City Labs), and a Prime 95B 25 mm CMOS camera (Photometrics) using a CFI Plan Apo Lambda 60x/1.4 oil or Plan Apo VC 100×/1.4 oil (Nikon). Images were captured using a penta dichroic 405/488/561/640/750 (Nikon), solid-state lasers 405 nm, 477 nm, 546 nm, and 638 nm and emission filters FF01-438/24, FF01-511/20, FF01-560/25, FF01-685/40 (Semrock), Nikon Multi-band for DAPI/Amylo-Glo, Alexa 488, Alexa 568, and Alexa 647, respectively. The data was captured with NIS-Elements software (v. 5.41.01 build 1709, Nikon). Whole-section tiled images were acquired with an Axioscan.Z1 slide scanner (Zeiss) at 20× magnification.

Human microglial GPC4 quantitation was performed blinded to clinical and pathological diagnosis. For each brain section tiled image, 10 random ROIs of the cortex were exported into FIJI [95]. IBA^+^ microglia were segmented using FIJI’s Trainable Weka Segmentation plugin [87] as previously described [96]. Briefly, the WEKA segmentation classifier was trained with 16 input 8-bit images (one image from each brain) using a fast random forest classifier with the following balanced training features: Gaussian blur, Hessian, membrane projections, Sobel filter, and difference of gaussians. The model was retrained for a total of 5 training sessions and then applied to each image to generate probability maps for microglia objects. The probability maps were thresholded to create microglia image masks. The image masks were then applied to the corresponding GPC4 channels to measure microglial GPC4 mean fluorescence across all brain sections. To quantify microglial GPC4 intensity as a function of distance from amyloid plaques in Alzheimer’s brains, eight Amylo-Glo-positive amyloid plaques were randomly chosen per brain section. For each plaque, IBA1^+^ microglia were defined as “near” if they were located within 125 μm from the center of the plaque. IBA1^+^ microglia were defined as “far” if they were located > 150 μm from the edge of all plaques but within 350 μm of the index plaque. Images for this analysis were 16-bit. To assess the relationship between microglial GPC4 and neuritic tau pathology, we stained tissue with α-GPC4 antibodies followed by BF-188, a conformation-sensitive fluorescent dye that differentially labels amyloid and tau pathology based on distinct spectral profiles. This enabled simultaneous visualization and spectral separation of plaques and tangles within the same tissue sections [97, 98]. Amyloid plaques were segmented using BF-188 signal and masks were generated by dilating each plaque boundary by 3 μm to capture peri-plaque regions. The integrated fluorescence intensity of tau pathology (AF546) and GPC4 (AF647) was then quantified on a per-plaque basis. For quantification of amyloid plaque and neurofibrillary tangle burden, whole brain section tiled images were analyzed using Zen 3.9 (Zeiss) and performed blinded to clinical and pathological diagnosis. The overlying neocortex was manually defined, and brightness thresholding was used to delineate anti-amyloid plaque and anti-CP13-positive areas. For each section, the total area of positive signal coverage was measured in the neocortex and expressed as percentage of total area analyzed in each brain [52].

### Mouse brain tissue and immunohistochemistry

Use of all animals was approved by the UVA Institutional and Animal Care and Use Committee (IACUC). All animals used in this study were handled according to IACUC approved protocols and housed in IACUC approved vivariums at the UVA MR-4. The 5xFAD [99] (Jackson Laboratory stock #34848), APP-SAA knock-in [100] (stock #034711), and C57BL/6J (stock #000664) mice were originally purchased from the Jackson Laboratory. All transgenic mice used were on a congenic C57BL/6 J background.

Samples from APP-SAA and matched C57BL/6J controls were collected at 9 months of age. Samples from 5xFAD and matched C57BL/6J control mice, all female, were collected at 8 months of age. Mice were anaesthetized with isoflurane and transcardially perfused with chilled PBS followed by chilled 4% PFA. Brains were post-fixed in 4% PFA on ice for 2 h before being transferred to 30% sucrose in PBS for cryoprotection. Frozen brains were coronally sliced at 30 μm on a cryostat (Leica CM1950).

For histological staining, free-floating sections were incubated with blocking solution (5% bovine serum albumin, 2% horse serum, 1% Triton X-100 in PBS) for two h at RT before staining. Primary and secondary antibodies were diluted in the same blocking solution. Slices were incubated in primary antibodies at 4 °C for 24 h and secondary antibodies at room temperature for 2 h. The following primary antibodies were used: rabbit anti-GPC4 (ProteinTech; 1:500); chicken anti-GFAP (Aveslabs; 1:500); and goat anti-IBA1 (Abcam; 1:300). The following secondary antibodies from Jackson ImmunoResearch were used at 1:100: anti-rabbit Cy3, anti-chicken AlexaFluor 488 (703-545-155), anti-goat Cy5 (711-175-147). After secondary antibody incubation, slices were stained with Amylo-Glo (Biosensis; TR-300-AG) according to manufacturer protocols in order to detect amyloid plaques, and then were mounted using Vectashield Plus mounting media (Vector Cat# H-1900). Slides were imaged using an Intelligent Imaging Innovations (3i) spinning-disc confocal microscope equipped with a Yokogawa CSX-X1 scan head using 40x and 63x objectives. Images were captured from the dentate gyrus and frontal cortex with a 63x objective for representative images and z-stacks captured with a 20x objective used for GPC4 quantification.

Image analysis was performed using Imaris software (Oxford Instruments, ver. 10.2.0). Microglia, astrocyte, and plaque surfaces were defined using automatic thresholds. After background subtraction, average intensity of the GPC4 channel within microglia and astrocyte surfaces was recorded.

### Drosophila immunohistochemistry and imaging

Flies were collected for staining at 10 days post eclosion (dpe). After CO_2_ anesthesia, heads were removed and fixed in 4% formaldehyde (Thermo Scientific) for 15 min at room temperature. Brains were dissected and incubated with primary antibodies dissolved in 0.3% Triton-X100 in PBS (PTX) for 48 h at 4 °C, followed by secondary antibodies in 0.3% PTX for 48 h at 4 °C. The following primary antibodies were used: chicken anti-GFP (Aves Labs, 1:1000); rat anti-mCherry (Invitrogen, 1:1000); mouse anti-amyloid (1:200, BioLegend), and rabbit anti-cDCP1 (Cell Signaling, 1:400). The following secondary antibodies from Jackson ImmunoResearch were used at 1:100: anti-rat Cy3, anti-chicken AlexaFluor 488, and anti-rabbit Cy5. After secondary antibody incubation, slices were stained with Amylo-Glo (Biosensis; TR-300-AG) according to manufacturer protocols, and then were mounted using Vectashield Plus mounting media (Vector; H-1900). Slides were imaged using an Innovative Imaging Innovations (3i) spinning-disc confocal microscope equipped with a Yokogawa CSX-X1 scan head using 40x and 63x objectives.

### Drosophila stocks

The following previously made *D. melanogaster* transgenes were used in this study: wrapper-Gal4DBD, Nrv2-VP16AD (CtxGlia-SplitGal4) [101], alrm-Gal4 [102], nSyb-QF2 [103], UAS-lacZ-NLS [104], QUAS-R-GECO1 [105], and stocks acquired from Bloomington Drosophila Stock Center (BDSC): UAS-dlpRNAi #1 (BDSC #34091), UAS-dlpRNAi #2 (BDSC #50540 [106]), UAS-dlp (BDSC #9160), QUAS-Aβ42 (BDSC #83347 [41]).

### Drosophila lifespan and climbing

*D. melanogaster* crosses were set on Molasses Formula Food (Archon Scientific) at 25 °C. Animals of the desired genotypes were collected at 0dpe and housed in vials containing corn syrup/soy medium (Archon Scientific) with 4–10 flies of the same sex per vial. The flies were assessed daily for viability, recorded, and remaining live flies were transferred to fresh vials every 2 days. Additionally, animals were tested for locomotion via climbing assay at 1 and 5dpe. Flies were transferred to empty 9 cm tall vials and allowed to habituate for 3 min. Vials were placed in a 9-vial holder and firmly tapped three times to knock all flies to the bottom of the vial. They were then rested for 30 s, tapped, rested again for 30 s, and received a final tap followed by the climbing assessment in which the highest height climbed by each fly in 10 s was recorded in cm. Each assay was performed between Zeitgeber time 8–9 and recorded on video. Analyses were performed using R, with lifespan analyzed with a Cox proportional hazard model with Bonferroni corrections and climbing data analyzed by a Tobit regression with Bonferroni corrections to account for floor and ceiling effects of the vial constraints.

### Phagocytosis assays

Microglia were plated at 50% confluence on Matrigel-coated 96-well plates. All phagocytosis assays were performed in quadruplicate using pHrodo-Red-labeled substrates. FL tau fibrils or magnetic beads were added to day 8 iTF Microglia at a final concentration of 100 nM or 1.92 × 10^7^ particles/mL, respectively. Phagocytosis was monitored every three hours for a duration of 24 h using an Incucyte SX5 (Sartorius). Four fields of view at 20x magnification per well were captured for each condition using phase and fluorescence channels. Incucyte 2023 A software equipped with the Cell-by-Cell module (Sartorius) was used to create image masks of both individual cells as well as phagocytosed substrates. Cellular integrated fluorescence intensity values of each well were averaged across treatment conditions and graphed as a function of time using Prism 10 (GraphPad). Where indicated, actin polymerization was inhibited by pretreating cells with 5 µM Cytochalasin D (Millipore Sigma; C8273) for 30 min before the addition of phagocytic substrates.

### Flow cytometry

iTF Microglia were differentiated at 15,000 cells per well in a 96-well plate. On day 8, they were treated with 1 µM Aβ_40_ or Aβ_42_ fibrils for 24 h. Cells were harvested with Versene for 8 min and resuspended and washed in DPBS plus 1% FBS, 1 mM EDTA, and 0.1% sodium azide. Immunostaining for cell surface proteins was performed at 4 °C for 1 h with α-10E4 (Amsbio,1:200) α-GPC4 (ProteinTech; 1:200), and α-GPC6 (Bioss; 1:200) antibodies followed by secondary staining at 4 °C for 1 h with Alexa Fluor 488 goat anti-mouse IgM or Alexa Fluor 647 goat anti-rabbit IgG (1:1000). For whole cell staining with GPC4 antibodies, 0.1% Saponin was added to cells for 15 min at RT prior to the addition of antibodies. For tau fibril cell surface binding experiments, cells were pretreated with 100 nM of anti-GPC4 sdAb-Fc for 24 h. Cells were then equilibrated for 4 °C for 15 min before the addition of tau fibrils-AF 647 for 60 min at 4 °C, washed with cold PBS, dissociated with Versene, and subjected to flow cytometry. Single cell fluorescence was measured using a Beckman Coulter CytoFlex flow cytometer. Median fluorescence intensity values of stained cells were normalized to vehicle-treated control samples or total GPC4 levels and plotted using Prism (GraphPad, v10).

For FRET flow cytometry assays, iPSC neuron biosensors were harvested with a 1:1 mix of papain (Worthington; LK003178) and Accutase (VWR / ICT; AT-104) for 10 min prior to resuspending in complete iNeuron media without phenol red. FRET flow cytometry was performed on a FACSCelesta (BD Biosciences). To measure mClover and FRET, cells were excited with the 488 nm laser, and fluorescence was captured with a 530/30 nm and 610/20 nm filter, respectively. To measure mApple, cells were excited with a 561 nm laser and fluorescence was captured with a 610/20 nm filter. To quantify FRET, we used a gating strategy similar to that previously described [52, 53]. The percentage of FRET-positive cells was used for all analyses. For each experiment, 10,000 cells per replicate were analyzed and each condition was analyzed in quadruplicate. Flow cytometry data were analyzed using FlowJo (v.10.9) and data were plotted using Prism (GraphPad, v10).

### GPC4 shedding assays

Conditioned media samples were harvested 24 h after Aβ_40/42_ fibril treatment and clarified by centrifugation (500 x g 5 min). Cells were lifted with Versene and pelleted by centrifugation (1000 x g 5 min). The pellet was resuspended in 100 µL of Luciferase assay buffer (Promega; N112A) to lyse and generate a 1× cell lysate sample. NanoLuc Furimazine substrate (Promega; N113A) was diluted 1:10,000 substrate to assay buffer and 1:4 in PBS. To minimize potential cross-luminescence, 20 µL of each sample was loaded into 384-well assay plate (Greiner) surrounded by empty wells. 60 µL of diluted Furimazine substrate was injected and luminescence of each well was read in a Tecan M1000 Pro. The average luminescence values of conditioned media or cell lysate samples were calculated and normalized to PBS-treated samples as a control. When normalizing to total GPC4 levels, the following equation was used: Normalized GPC4 = Shed GPC4 / (Shed GPC4 + Cell Lysate GPC4). For shedding inhibition assays, Marimastat was added to cells one hour prior to the addition of Aβ fibrils.

### Protein pull downs

Tau-biotin (1 µg) was mixed with 3 µg of mammalian-derived GPC4 WT (Acro), 3 µg of mammalian-derived lipidated APOE3, or both in a total of 50 µL of 100 mM NaCl, 10 mM HEPES containing 0.05% Tween20. The proteins were incubated O/N at 4 °C while mixing at 700 RPM. The next day, the protein complexes were added to 50 µL of washed streptavidin Magnesphere Paramagnetic Beads (Promega; Z5482) and incubated for 1 h at RT with mixing at 700 RPM. The magnetic beads were then washed three times with TBS-T containing 0.5 M NaCl and eluted in 10 µL of 1 M glycine, pH 2 for 10 min at RT.

### Biolayer interferometry

We characterized the interactions of tau with GPC4 and APOE3 via biolayer interferometry (Octet RED384, FortéBio). Assay buffer was composed of 10 mM HEPES, pH 7.4, 0.1% BSA and 0.01% Tween 20 in deionized water. Biotinylated tau-441 (rPeptide) was diluted with assay buffer to a concentration of 100 nM and Glypican 4 and APOE3 was diluted to a final concentration of 250 nM. In addition, a control anti-tau antibody, MD3.1 [107], was diluted in assay to a final concentration of 50 nM. Streptavidin biosensors (FortéBio) were equilibrated in 200 µL of assay buffer for 30 min before use in a 96-well plate (Greiner). For experimental analysis, 80 µL of tau-biotin, Glypican 4, APOE3, and MD3.1 were dispensed into respective wells on a 384-well assay plate. The sensors were loaded with tau-biotin, followed by quenching of the remaining streptavidin on the sensors with 10 µM biotin buffer (assay buffer with 50 mM biotin), an association step (1800 s) and a dissociation step (800 s). The assay was performed at 25 °C with 1000 rpm shaking. Data were processed and analyzed using the Octet 12.2.2.4 data analysis software. Graphs were subsequently generated in GraphPad Prism.

### Negative stain transmission electron microscopy

Fibrillization reactions were examined for the presence of filaments by negative staining and transmission electron microscopy (TEM). Briefly, 5 µL of sample was spotted on a glow-discharged carbon/formvar-coated 300-mesh grid for 1 min. Excess liquid was blotted off with Whatman paper, followed by washing with another 5 µL of ddH_2_O. Excess liquid was removed by blotting and the grid was stained with a 2% uranyl acetate solution for 1 min. Excess liquid was blotted off and the grid was imaged by TEM on an FEI Tecnai G2 Spirit Biotwin operating at 120 kV. qPCR. RNA from mammalian cells were extracted and purified using Tri-isolate RNA Pure kits (IBI Scientific; IB47632) according to manufacturer guidelines. For *Drosophila* mRNA, flies were collected at 10 days post eclosion and flash frozen. Forty heads, mixed male and female, were removed and pooled per sample. Samples were homogenized and RNA was isolated using a Qiagen RNeasy kit. For qPCR assays, RNA was DNAse-treated and converted to cDNA using Quantitect Reverse-Transcription kit (Qiagen; 205311). qPCR reactions were performed using SYBR Select Master Mix (Thermo Scientific; 4472908). Final primer concentrations were 250 nM and Tm was 60 °C. Fluorescent emissions were detected using Bio-Rad CFX Connect qPCR instrument. Data were analyzed using ΔΔCT method. For representation of the data in figures, a Z-score was computed and heatmaps were generated with heatmapper.ca. qPCR primers are provided in Supplementary Material 16.

### Lentiviral transduction of iPSCs with sgRNA constructs

Pooled sgRNAs were lentivirally packaged in HEK293T cells (ATCC; CRL-3216) as previously described [83] using TransIT Lenti Reagent (Mirus Bio; MIR 6600), and introduced into CRISPRi or CRISPRa iPSCs. Cells were selected with 1 µg/mL puromycin (Gibco; A11138-03) for 7 d after which cells were cultured for 2–4 d in the absence of puromycin to allow them to recover. sgRNA protospacer sequences are provided in Supplementary Material 16.

Statistics. All cell culture data are expressed as mean ± S.E.M. from 3 or more independent experiments, and the level of significance between 2 groups was assessed with Student’s t-test. For experiments consisting of more than 2 groups, one-way or two-way ANOVA followed by Holm-Sidak test was applied. A value of *p* < 0.05 was considered statistically significant.

### Antibodies

Anti-10E4 mouse primary antibody (Amsbio; F58-10E4).

Anti-Amyloid mouse primary antibody (Millipore Sigma; MAB5206).

Anti-Amyloid mouse primary antibody (BioLegend; SIG-39320).

Anti-cleaved Dcp-1 antibody (Cell Signaling Technology; 9578).

Anti-Glypican 1 rabbit primary antibody (Abcam; EPR19285).

Anti-Glypican 2 rabbit primary antibody (Invitrogen; PA5-115299).

Anti-Glypican 3 mouse primary antibody (Invitrogen; MA5-17083).

Anti-Glypican 4 rabbit primary antibody (ProteinTech; 13048-1-AP).

Anti-Glypican 5 mouse primary antibody (R&D Systems; 297716).

Anti-Glypican 6 rabbit primary antibody (Bioss Antibodies; bs-2177R).

Anti-IBA1 rabbit primary antibody (Cell Signaling Technology; 17198).

Anti-IBA1 guinea pig primary antibody (Synaptic Systems; 234 308).

Anti-PU.1 rabbit primary antibody (Cell Signaling Technology; 2266).

Anti-Tau mouse primary antibody (CP13; gift from Peter Davies).

Anti-TMEM119 mouse primary antibody (BioLegend; 853301).

Alexa Fluor 488 donkey anti-chicken IgG secondary antibody (Jackson ImmunoResearch; 703-545-155).

Alexa Fluor 488 goat anti-guinea pig IgG secondary antibody (Invitrogen; A-11073).

Alexa Fluor 488 Plus goat anti-mouse IgG secondary antibody (Invitrogen; A32723).

Alexa Fluor 488 goat anti-mouse IgM secondary antibody (Invitrogen; A-21042).

Alexa Fluor 546 goat anti-rabbit IgG secondary antibody (Invitrogen; A-11035).

Alexa Fluor 647 goat anti-rabbit IgG secondary antibody (Invitrogen; A-21245).

Biotinylated horse anti-mouse IgG secondary antibody (Vector; BA-2000-1.5).

Cy3 donkey anti-rabbit IgG secondary antibody (Jackson ImmunoResearch; 711-165-152).

Cy3 donkey anti-rat IgG secondary antibody (Jackson ImmunoResearch; 712-165-153).

Cy5 donkey anti-goat IgG secondary antibody (Jackson ImmunoResearch; 705-175-147).

Cy5 donkey anti-rabbit IgG secondary antibody (Jackson ImmunoResearch; 711-175-152).

## Supplementary Information

Below is the link to the electronic supplementary material.

Supplementary Material 1

## Data Availability

All data generated in this study are included in this published article. The mass spectrometry proteomics data have been deposited to the ProteomeXchange Consortium via the PRIDE partner repository with the dataset identifier PXD060977 [108].

## Abbreviations

Aβ: Amyloid-beta
AD: Alzheimer’s disease
APOE: ApolipoproteinE
CRISPRi: Clustered regularly interspaced short palindromic repeats interference
CRISPRa: Clustered regularly interspaced short palindromic repeats activation
FRET: Förster resonance energy transfer
GPC4: Glypican 4
GPI: Glycosylphosphatidylinositol
HSPG: Heparan sulfate proteoglycan
IL-34: Interleukin 34
iPSC: Induced pluripotent stem cells
iTF Microglia: Induced transcription factor microglia
LPS: Lipopolysaccharide
M-CSF: Macrophage colony-stimulating factor
MMP: Matrix metalloprotease
NaN_3_: Sodium azide
NLuc: NanoLuc luciferase
qPCR: Quantitative polymerase chain reaction
RNAi: RNA interference
TGF-β1: Transforming growth factor beta 1
TREM2: Triggering receptor expressed on myeloid cells 2
WT: Wild type
